# Copper-Containing Nanoparticles and Organic Complexes: Metal Reduction Triggers Rapid Cell Death via Oxidative Burst

**DOI:** 10.3390/ijms222011065

**Published:** 2021-10-14

**Authors:** Sergey A. Tsymbal, Anna A. Moiseeva, Nikol A. Agadzhanian, Svetlana S. Efimova, Alina A. Markova, Dmitry A. Guk, Olga O. Krasnovskaya, Victoria M. Alpatova, Andrei V. Zaitsev, Anna V. Shibaeva, Victor V. Tatarskiy, Marina S. Dukhinova, Valentina A. Ol’shevskaya, Olga S. Ostroumova, Elena K. Beloglazkina, Alexander A. Shtil

**Affiliations:** 1 International Institute of Solution Chemistry of Advanced Materials and Technologies, ITMO University, 197101 Saint Petersburg, Russia; agadzhanian@scamt-itmo.ru (N.A.A.); dukhinova@scamt-itmo.ru (M.S.D.); 2 Department of Chemistry, Moscow State University, 119991 Moscow, Russia; moiseeva@org.chem.msu.ru (A.A.M.); dmh200949@gmail.com (D.A.G.); krasnovskayao@gmail.com (O.O.K.); beloglazki@mail.ru (E.K.B.); shtilaa@yahoo.com (A.A.S.); 3 Institute of Cytology, Russian Academy of Sciences, 194064 Saint Petersburg, Russia; efimova@incras.ru (S.S.E.); osostroumova@mail.ru (O.S.O.); 4 Emanuel Institute of Biochemical Physics, Russian Academy of Sciences, 119334 Moscow, Russia; alenmark25@gmail.com (A.A.M.); anna-shiba@mail.ru (A.V.S.); 5 A.N.Nesmeyanov Institute of Organoelement Compounds, Russian Academy of Sciences, 119334 Moscow, Russia; vika.alpatova@gmail.com (V.M.A.); porphyrin@yandex.ru (A.V.Z.); olshevsk@ineos.ac.ru (V.A.O.); 6 Institute of Gene Biology, Russian Academy of Sciences, 119334 Moscow, Russia; tatarskii@gmail.com; 7 Blokhin National Medical Research Center of Oncology, 115478 Moscow, Russia

**Keywords:** copper, reactive oxygen species, redox potential, drug resistance, cell death, tumor cells

## Abstract

Copper-containing agents are promising antitumor pharmaceuticals due to the ability of the metal ion to react with biomolecules. In the current study, we demonstrate that inorganic Cu^2+^ in the form of oxide nanoparticles (NPs) or salts, as well as Cu ions in the context of organic complexes (oxidation states +1, +1.5 and +2), acquire significant cytotoxic potency (2–3 orders of magnitude determined by IC_50_ values) in combinations with N-acetylcysteine (NAC), cysteine, or ascorbate. In contrast, other divalent cations (Zn, Fe, Mo, and Co) evoked no cytotoxicity with these combinations. CuO NPs (0.1–1 µg/mL) together with 1 mM NAC triggered the formation of reactive oxygen species (ROS) within 2–6 h concomitantly with perturbation of the plasma membrane and caspase-independent cell death. Furthermore, NAC potently sensitized HCT116 colon carcinoma cells to Cu–organic complexes in which the metal ion coordinated with 5-(2-pyridylmethylene)-2-methylthio-imidazol-4-one or was present in the coordination sphere of the porphyrin macrocycle. The sensitization effect was detectable in a panel of mammalian tumor cell lines including the sublines with the determinants of chemotherapeutic drug resistance. The components of the combination were non-toxic if added separately. Electrochemical studies revealed that Cu cations underwent a stepwise reduction in the presence of NAC or ascorbate. This mechanism explains differential efficacy of individual Cu–organic compounds in cell sensitization depending on the availability of Cu ions for reduction. In the presence of oxygen, Cu^+1^ complexes can generate a superoxide anion in a Fenton-like reaction Cu^+1^L + O_2_ → O_2_**^−.^** + Cu^+2^L, where L is the organic ligand. Studies on artificial lipid membranes showed that NAC interacted with negatively charged phospholipids, an effect that can facilitate the penetration of CuO NPs across the membranes. Thus, electrochemical modification of Cu ions and subsequent ROS generation, as well as direct interaction with membranes, represent the mechanisms of irreversible membrane damage and cell death in response to metal reduction in inorganic and organic Cu-containing compounds.

## 1. Introduction

Biocompatible agents containing copper in an inorganic form or in the context of metal–organic complexes are widely used in a variety of applications, largely as antibacterial and antifungal drugs, as well as in tumor treatment [1,2,3,4,5]. Copper oxide-based materials varying from sub-10 nm to 40–60 nm (nanoparticles; NPs) and fine (<10 µM) particles have demonstrated cytotoxicity against prokaryotic, yeast, mammalian cell lines and zebrafish embryos [6,7,8,9]. Coordination of Cu^2+^ or Cu^1+^ with complex organic scaffolds has yielded a number of perspective multi-targeting chemotypes with differential antitumor properties [10,11,12,13,14]. Thus, copper has proved its therapeutic value in a wide variety of applications.

Copper’s unique sensitivity to the electrochemical status makes it particularly advantageous in situations where the biological effects are mechanistically dependent on reactive oxygen species (ROS) [15,16,17,18,19]. These studies have highlighted the opportunity of using Cu-containing compounds alone or in combinations with conventional and novel agents. However, undesired toxicity of metal-containing drugs may limit their practical use. Zheng et al. [20] showed that the combination of CuCl_2_ and the antioxidant N-acetylcysteine (NAC) induced a sizeable cytotoxic effect in cancer cell lines via the production of hydroperoxide. We are interested in determining whether Cu-containing NPs or Cu–organic complexes at non-toxic concentrations become potent against tumor cells if combined with non-toxic doses of NAC. Here, we demonstrate that these combinations triggered oxidative stress within the initial few hours followed by a complex mode of cell death regardless of the species and tissue origin. The effect of potentiation was limited to Cu-containing compounds since NAC did not change the cytotoxicity of other tested metals. Importantly, electrochemical experiments showed that the ability to synergize with Cu-containing compounds was attributed not to the antioxidant property of NAC but to a rapid reduction of Cu^2+^ to Cu^1+^. This mechanism was demonstrated for CuO NPs and Cu-coordinated organic complexes and represents a means of copper reduction in inorganic and organic compounds as an approach to oxidative burst-mediated purge of pleural or abdominal cavities from metastatic tumor cells.

## 2. Results

### 2.1. Synthesis and Characterization of CuO NPs

**Figure 1:**
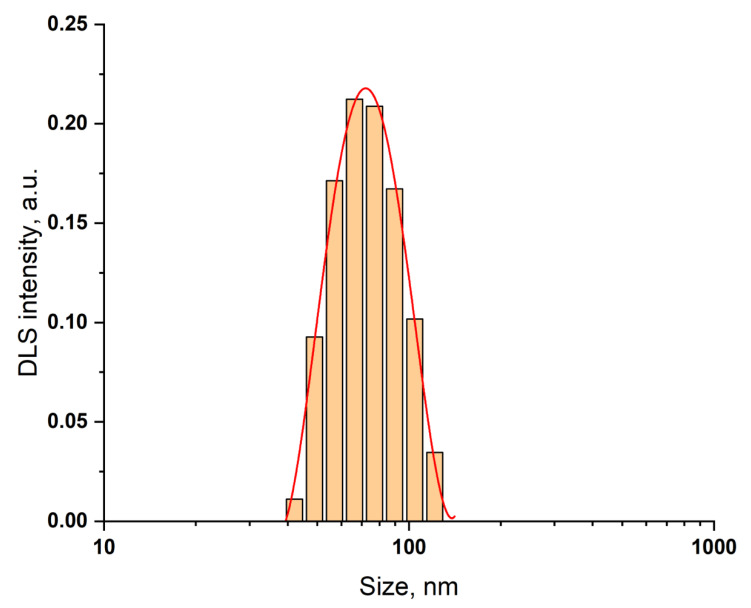
Size distribution of CuO NPs determined by DLS.

CuO NPs were synthesized by the precipitation method [21]. The size of NPs determined by DLS was 80 ± 20 nm (Figure 1); zeta potential was +15 ± 0.5 mV. The properties of magnetite and ZnO NPs have been reported by us [22].

### 2.2. NAC Dramatically Increases the Cytotoxicity of CuO NPs, Leading to Rapid Cell Death

CuO NPs alone were moderately cytotoxic against a panel of mammalian cells as determined in MTT assays after 72 h exposure (Table 1). Given that the cytotoxicity of copper-containing compounds has been mechanistically attributed to ROS [23], we used non-toxic concentrations of NAC to modulate the efficacy of CuO NPs. Importantly, NAC (1 mM) dramatically decreased cell survival in combination with 1 µg/mL CuO, the concentration that corresponds to ~IC_5_ if added alone. Table 1 shows that the combination of CuO + NAC caused remarkable sensitization of all tested cell lines, including the sublines with molecular determinants of antitumor drug resistance, such as *MDR*1/P-glycoprotein for K562/4 subline, anti-oxidative systems for SCOV3/CDDP subline, and an altered response to doxorubicin (Dox) due to p53 deficiency in the HCT116p53KO subline [24,25] (and the refs therein). Fold sensitization (determined as the ratio of IC_50CuO_/IC_50CuO+NAC_) achieved values as high as 2–3 orders of magnitude (Table 1).

**Figure 2:**
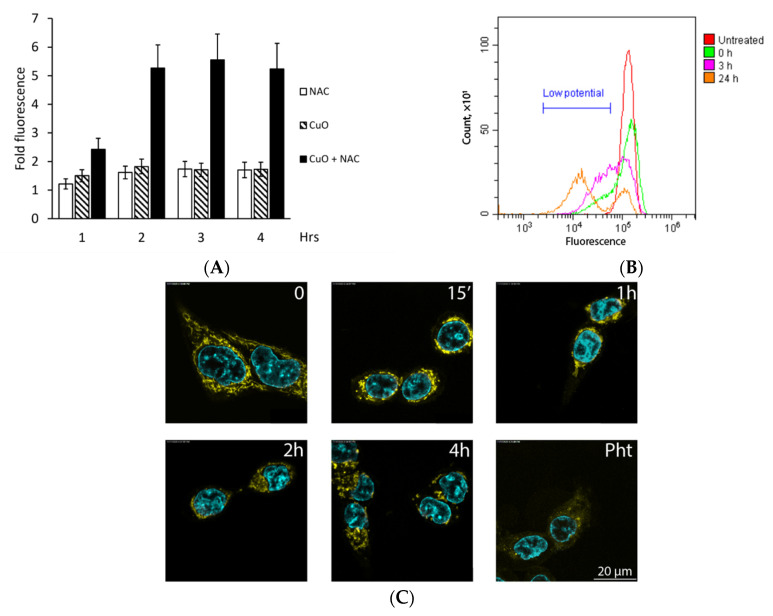
ROS burst and mitochondrial responses upon treatment with the combination of 1 μg/mL CuO and 1 mM NAC. (**A**,**B**): K562 cells; *C*: HCT116 cells. (**A**) Carboxy-H_2_DCFDA (5 µM) was added 30 min before the addition of the combination. Cell-associated fluorescence was measured in the FITC channel. Mean fluorescence intensity in untreated (no combination, carboxy-H_2_DCFDA only) cells was taken as 1. Data are mean+SD of three experiments. (**B**) Time-dependent decrease in mitochondrial electric potential (shown with the low potential marker). (**C**) Cells were labeled with the ROS indicator DHR 123 (yellow) and the nuclear stain Hoechst 33258 (blue) and analyzed by laser confocal microscopy. Pht, photolon.

For studies of cell death mechanisms, we used 0.1–1 µg/mL CuO and 1 mM NAC. We investigated whether CuO + NAC can trigger intracellular oxygen burst. Figure 2A shows that CuO (1 µg/mL) or NAC (1 mM) alone evoked minor changes in fluorescence of the intracellular ROS probe DCFDA. In contrast, by 1 h of cell exposure to the combination of CuO and NAC, a statistically significant increase in DCFDA fluorescence was detectable; its maximum was reached by 2 h and remained sustained for at least 4 h (Figure 2A). These effects were paralleled by the time-dependent decrease in mitochondrial membrane electric potential (Figure 2B). Of note, a population with a lowered potential was detectable by 1 h of treatment of K562 cells with CuO + NAC (not shown).

To gain insight into the morphology of cell damage, we used the adherent HCT116 cell line. In untreated cells, the mitochondria stained with DHR 123 were visualized as definitive rod-shaped cytoplasmic structures (Figure 2C, upper left panel). In striking contrast, treatment with CuO NPs and NAC for only 15 min led to loss of the regular mitochondrial structure; during longer exposure to the combination, the mitochondria were detectable largely in the perinuclear area but less so across the cytoplasm (Figure 2C). By 4 h, the cytoplasm with mitochondria and the nuclei were disintegrated. Interestingly, the shape of the nuclei as well as the chromatin density remained unaltered, indicating direct damage of the cytoplasmic content as a result of the combination of CuO NPs and NAC. For comparison, laser illumination of cells loaded with the reference compound photolon (Pht), a chlorin e_6_ derivative, led to dramatic damage of the cytoplasm (note the clusters of fragmented organelles and a significant decrease in DHR fluorescence in Figure 2C, bottom right panel). No mitochondrial changes were induced by either CuO or NAC alone (not shown).

**Figure 3:**
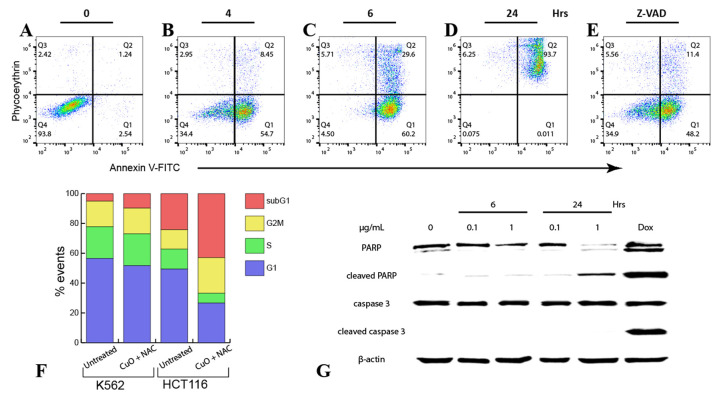
Death-associated events in cells treated with CuO NPs + NAC. The K562 (**A**–**G**) or HCT116 (**F**) cells were treated with 1 μg/mL CuO and 1 mM NAC for up to 24 h in the absence (**A**–**D**,**F**) or presence (**E**) of the pan-caspase inhibitor z-VAD (50 μM, 4h), then washed and stained with annexin V-FITC/PI (**A**–**E**) or lysed in the PI/cell cycle buffer (**F**) followed by flow cytometry. (**G**) Immunoblotting with K562 cells. See the Materials and Methods (Section 4) and text for details.

ROS generation and the decrease in mitochondrial electric potential were concomitant with other parameters of cell death. Approximately 50% of K562 cells became annexin V positive by 4 h of treatment with 0.1 μg/mL CuO + 1 mM NAC (Figure 3A,B). By 6 h, a population of annexin V/PI double-positive cells was detectable; after 24 h, virtually all cells were stained with annexin V and PI (Figure 3C,D). Interestingly, DNA fragmentation determined as the percentage of subG1 events was rather minor (Figure 3F), suggesting that, in K562 cells, death by the combination of CuO and NAC was largely associated with damage of the plasma membrane. This conclusion was supported by the lack of effect of the pan-caspase inhibitor z-VAD on annexin V/PI reactivity (Figure 3E).

To evaluate the plasma membrane integrity in HCT116 cells treated with CuO + NAC, we used laser confocal microscopy. Pht served as a reference compound since photoactivation of tetrapyrrolic compounds is known to trigger primary loss of plasma membrane integrity [26,27]. Accordingly, microscopic laser illumination of Pht loaded cells during imaging resulted in PI influx within the initial 3–5 min (Figure S1). In contrast, PI staining of cells treated with 1 µg/mL CuO together with 1 mM NAC developed slower; however, by 2 h, intracellular PI fluorescence was detectable (Figure S1), indicating rather fast damage of the plasma membrane. No PI entry was detectable after the addition of CuO or NAC alone (not shown). Next, activation of caspase 3 was determined by neither by immunoblotting (Figure 3G) nor immunostaining (Figure S2). No proteolytic processing of poly(ADPribose)polymerase (PARP) was observed upon cell exposure to CuO + NAC, whereas the reference compounds Dox or PF-114, a Bcr-Abl inhibitor [28], induced cleavage of PARP and caspase 3 in K562 cells (Figure 3G and Figure S2). Altogether, we interpreted the complex mode of cell death by CuO + NAC as a caspase-independent apoptosis coupled with an early necrosis.

### 2.3. A Combination of Copper-Containing Compounds and the Reducing Agent Is the Requirement for Cell Sensitization

The above experiments demonstrated the cytotoxic potency of the combination of CuO NPs and NAC. We were interested in determining whether cell sensitization can be accomplished with different agents (inorganic or organic) that contain copper or other metals. In addition, we aimed to answer the following research question: can α-tocopherol or ascorbate replace NAC in combinations? The efficacy of sensitization by NAC was similar for acetate and copper oxide (Figure S3). In contrast, no sizeable sensitization by NAC was achieved with Fe, Mo, Zn, or Co oxides, indicating that Cu is the preferred metal for this combination. A-Tocopherol had no effect, whereas sodium ascorbate (80 µM) significantly potentiated the cytotoxicity of CuO NPs (Figure S3). Furthermore, cysteine demonstrated similar sensitizing potency to that of NAC, whereas neither methionine nor phenylalanine changed CuO cytotoxicity (Figure S3). Thus, copper (either in the oxide or as free ions) and an appropriate reducing agent are necessary for the efficient cytotoxic combination.

**Scheme 1:**
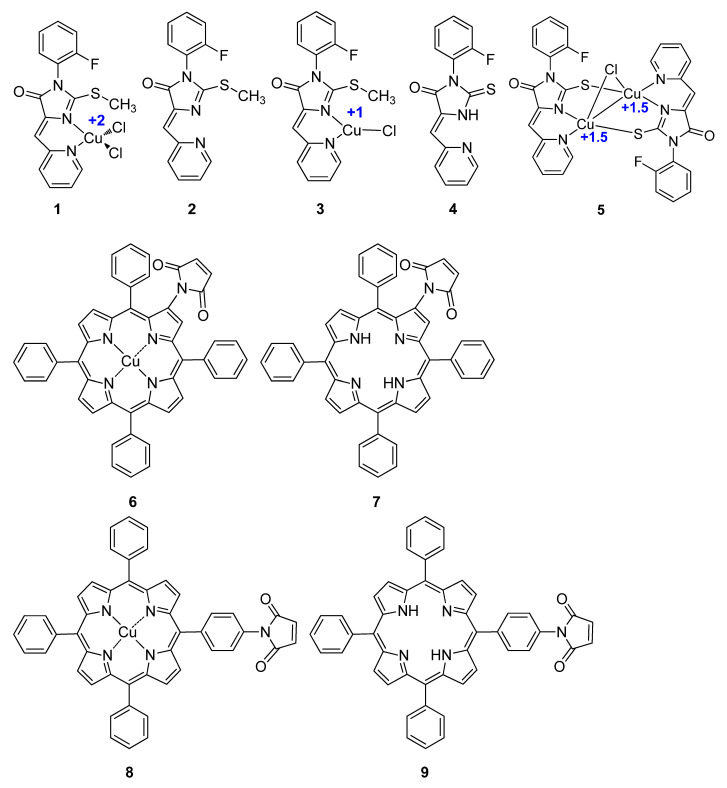
Structures of copper organic complexes and the respective metal-free ligands used in the study.

Next, we investigated the role of Cu ion reduction in the context of the organic complexes. We have previously reported a series of biologically active Cu–organic complexes [10,29]. In the present study, we tested the complexes of copper and 5-(2-pyridylmethylene)-2-thio-imidazol-4-one (compounds **1**, **3, 5**) with different oxidation states of Cu atoms (+1, +1.5, +2), as well as the respective metal-free organic ligands **2** and **4**. Furthermore, we studied Cu-porphyrin complexes **6**, **8,** and the metal-free carcasses **7** and **9** in combinations with 1 mM NAC (Scheme 1). As shown in Table 2 and Figure S4, NAC substantially increased the cytotoxic potency of Cu complexes **1** and **3,** whereas in cases of metal-free **2** and **4**, sensitization in the presence of NAC was much less pronounced. Similar results were obtained with combinations of NAC and Cu porphyrins **6** and **8** vs. metal-free counterparts **7** and **9**. Interestingly, fold sensitization for individual Cu organic compounds varied significantly, with a maximum value of 364.0 achieved for **1** (Table 2). Compounds **2**, **7** and **9** were virtually non-toxic; their IC_50_ values were not determined. NAC had no effect on the viability of HCT116 cells in combinations with **2, 7** and **9**.

These results strongly suggested that the context for the copper ion, particularly its accessibility to the reducing agent, is critical for the efficacy of cell killing. We used cell-free systems to gain insight into the mechanisms of copper–NAC interaction.

### 2.4. Electrophysiological Measurements of Membrane Conductance in the Presence of CuO NPs and NAC

**Figure 4:**
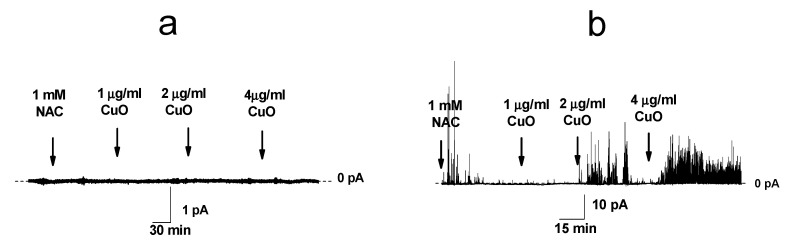
Examples of current fluctuations induced by CuO NPs (1–4 µg/mL) and NAC (1 mM) in lipid bilayers composed of POPC (**a**) and POPG (**b**) and bathed in 0.1 M KCl pH 7.4. The transmembrane voltage was 50 mV. Arrows indicate the addition of NAC or CuO NPs into the membrane bathing solution. One experiment out of three replicates with essentially the same results is shown.

To investigate the influence of CuO NPs and NAC on the permeability of model lipid membranes, the effects on the ion conductance of planar lipid bilayers made from neutral and negatively charged phospholipids were tested. CuO NPs (1–4 µg/mL) (Figure S5a) and NAC (1 mM), alone or in combination (Figure 4a), had no effect on the ion current fluctuations through electrically neutral POPC bilayers. Furthermore, CuO NPs did not change the conductance of negatively charged POPG membranes (Figure S5b). In contrast, NAC alone triggered brief transmembrane current fluctuations in POPG bilayers. This effect was further augmented by the addition of CuO NPs, but at relatively high concentrations (>2 µg/mL) (Figure 4b).

### 2.5. Leakage of the Fluorescent Dye from Liposomes Modified by NAC and CuO NPs

**Figure 5:**
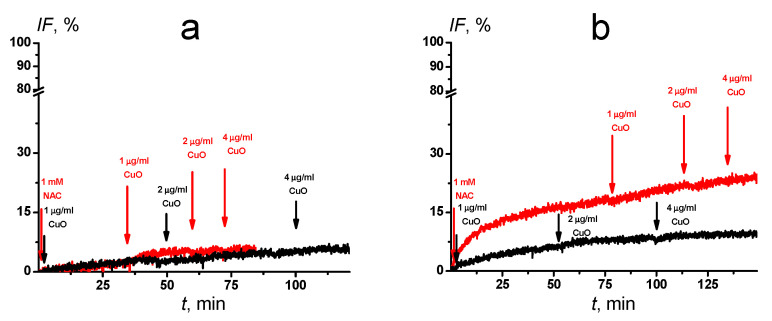
Time dependence of relative fluorescence of calcein (*IF*, %) leaked from POPC (**a**) and POPG (**b**) vesicles. CuO NPs alone (black curves) or in combination with 1 mM NAC (red curves) were added to the liposomal suspension at indicated concentrations (arrows). One representative experiment out of three independent measurements is shown.

Next, we investigated whether CuO NPs, NAC, or their combinations can damage the membranes of unilamellar lipid vesicles by triggering the release of the fluorescent dye calcein. Only a minor leakage of calcein from neutral POPC liposomes was detectable in the presence of CuO NPs alone or with NAC (IF ~ 6–8%; Figure 5a). However, unlike CuO NPs (Figure 5a, black curve) resulting in less than 10% marker leakage, NAC alone induced calcein release from negatively charged POPG liposomes (IF = 18%), and this effect was slightly potentiated by 1–4 µg/mL CuO NPs (IF ~ 25%; Figure 5b, red curve). These results suggested that NAC directly interacts with negatively charged POPG.

### 2.6. DSC of Liposomal Suspensions

To investigate the effects of CuO NPs and NAC on lipid packing, we performed DSC of liposomes composed of neutral (DPPC) and negatively charged (DPPG) phospholipids with saturated acyl chains. Figure S5 and Table S1 show thermodynamic parameters of DPPC and DPPG liposomes treated with CuO NPs and/or NAC. Each treatment alone or in combination evoked no noticeable changes in thermotropic behavior of neutral DPPC (Figure S6a,c,e). Moreover, CuO NPs alone did not affect the endotherm of negatively charged DPPG (Figure S6d). In contrast, NAC significantly altered the thermodynamic parameters of DPPG—the main peak corresponding to acyl chain melting was shifted to higher temperatures (~1 °C; Figure S6b), and a deconvolution of the peak was detectable (Figure S6b, inset). The latter observation indicates the presence of lipid phases that varied in the content and structure due to demixing the lipid and NAC. This effect was further augmented by the addition of CuO NPs—the maximum of main transition was shifted (>2 °C) toward higher temperatures (Figure S6f). The main peak deconvolution was enlarged, demonstrating at least three lipid phases (Figure S6f, inset).

### 2.7. Electrochemical Monitoring of Copper Redox States in the Presence of NAC or Ascorbate 

**Figure 6:**
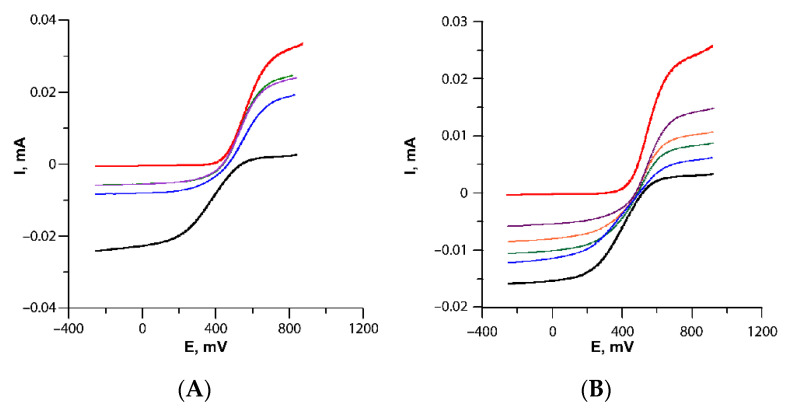
Voltammograms (RDE) of Cu^+2^ reduction in complex **1**. (**A**) Mixtures of **1** and NAC in a 1:1 ratio registered successively within 25 min after mixing the reagents (blue, violet, and green curves); registered 5 min after mixing of **1** and NAC in a 1:3 ratio (red). (**B**) Mixtures of **1** and ascorbate in a 1:1 ratio registered successively within 100 min after mixing the reagents (blue, orange, green, and violet); registered 40 min after mixing **1** and ascorbate in a 1:3 ratio (red). Black, compound **1** alone.

To test the possibility of Cu^2+^ reduction, we studied the reactions of the reducing agents NAC or ascorbate with CuO NPs, as well as with organic complex **1** (Figure 6), which contained one copper ion at Cu^+2^ oxidation state. Reactions were assessed by cyclic voltammetry (CV) and rotating disk electrode (RDE) techniques.

The RDE voltammogram allows one to identify the redox transition states of metals. For Cu^+1^-containing compounds, only the oxidation current was observed (anodic process Cu^+1^ → Cu^+2^), whereas for Cu^+2^-containing compounds, the reduction current was observable (cathodic reaction Cu^+2^ → Cu^+1^). For mixtures containing Cu^+1^/Cu^+2^, the voltammograms showed both anodic and cathodic currents. Voltammograms were recorded at 960-250 mV, corresponding to a Cu^+2^–Cu^+1^ transition in **1**.

In RDE experiments within this range of electric potential, the solution of **1** (Cu^2+^) revealed only a cathode wave (negative current values) corresponding to the electrochemical reduction of LCu^2+^ → LCu^1+^ (Figure 6A,B, black curves). When NAC or ascorbate was added (1:1 molar ratio), the cathodic current decreased, while the anodic current increased in terms of the voltammograms recorded after 5 min, which indicates a chemical reduction of Cu^2+^ to Cu^1+^ by NAC or ascorbate (Figure 6A,B, blue, green, orange, and violet curves). The value of the cathodic current reflects the fraction of the non-reduced (initial) complex LCu^+2^ in the solution, whereas the value of the anodic current reflects the portion of the reduced LCu^+1^ at a particular time.

In the reaction of **1** with NAC at 1:1, the equilibrium was established after 25 min at an anodic current/cathodic current ratio of 5:1. With a threefold excess of NAC, a wave with completely positive current values was observed after 5 min, which corresponds to the oxidation process formed in the solution of the complex with monovalent copper LCu^+1^ → LCu^+2^, where L is the organic ligand (Figure 6A, red curve).

When **1** interacted with ascorbate in a 1:1 ratio, the reduction of Cu^+2^ to Cu^+1^ in the complex proceeded slower; an anodic current/cathodic current equilibrium 3:1 was established after 100 min. With an ascorbate/**1** ratio of 3:1, the full LCu^+2^ reduction occurred within 40 min (Figure 6B). In the reduction of Cu^+2^ into Cu^+1^, NAC oxidation is a one-electron process, and ascorbate oxidation is a two-electron process; nevertheless, reduction of Cu^+2^ with ascorbate proceeded slower than reduction by NAC. Apparently, differential reaction rates in the presence of NAC or ascorbate indicate different mechanisms of oxidation of individual reducing molecules. Thus, NAC and ascorbate at a threefold excess completely reduced Cu^+2^ to Cu^+1^ in the solution of **1** within a short time.

The Cu^+2^ to Cu^+1^ reduction in the presence of NAC was observed using RDE. For the suspension of CuO NPs, this process was studied in a dimethylformamide/water mixture. However, in this heterogenous system, the effect was less noticeable due to a low intensity of anodic and cathodic currents.

### 2.8. Analysis of Cu^+2^/NAC Interaction by Mass Spectroscopy

To test the hypothesis about the participation of NAC in the redox reaction with copper(+2), we carried out an investigation of the reaction mixture that formed when mixing the indicated reducing agent with complex **1**, in which the formation of a copper(+1) complex was determined by the RDE examination. The two main peaks in the LC-MS mass spectra of the mixture obtained by the reaction of **1** with NAC are attributable to the initial ligand and to the oxidation product of NAC to the corresponding disulfide (a peak with a mass of 325 in positive ions and 323 in negative ions; Figures S7 and S8).

**Scheme 2:**
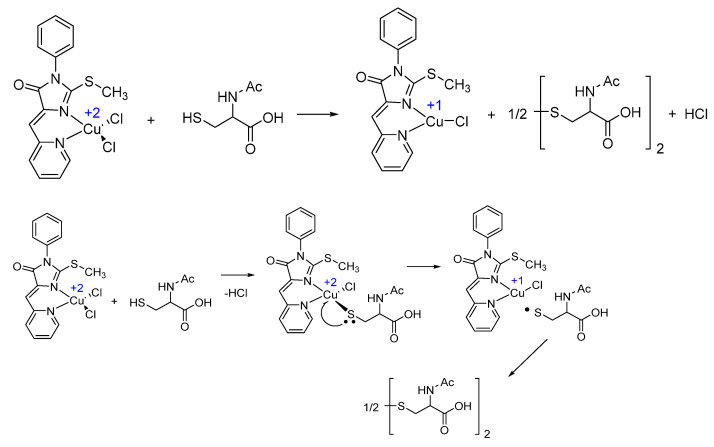
Molecular transformations during stepwise reduction of Cu^2+^.

No copper-containing compounds were detected by mass spectroscopy; the reduction products and the initial complex are likely to fall apart on the column, which is often observed for coordination compounds. Thus, the reaction of the Cu^+2^ complex with NAC can be represented as shown in Scheme 2, top panel. Most likely, the reaction proceeded in the coordination sphere of copper with the initial substitution of thiol group for one of the chloride anions followed by intramolecular electron transfer from S to Cu atoms, elimination of the sulfur-centered radical from copper, and dimerization of this radical to disulfide (Scheme 2, bottom panel).

It is also possible, although less likely, that the reaction proceeded intermolecularly without intermediate entry of NAC into the coordination sphere of the copper atom. Thus, NAC oxidation was observed to occur upon interaction with Cu^2+^-containing complex **1**. The sulfur atoms became no longer available for copper reduction as determined by disappearance of SH groups (detectable with the Ellman’s reagent; Figure S9) upon the addition of NAC to CuO NPs.

Finally, LC-MS analysis of the reaction mixture, NAC–CuO NPs, demonstrated essentially the same results as those of NAC + complex **1**. At a molar ratio of NPs/NAC 1:1 (No. 1), the intensity of the peak 325 (disulfide) was only minor, whereas at a molar ratio 1:3 (No. 2), this value was 50%. If NPs/NAC ratio molar was 1:10 (No.3), peak 325 was 4%, whereas the major (80%) peak was attributed to mass 437 (disulfide 325 + copper 64 + CH_2_SH 49 (Figures S10 and S11)). These results indicated that at a 10-fold NAC excess, all of the copper was in an ionic form, and these ions were coordinated by the products generated in the process of reduction.

## 3. Discussion

We demonstrated that the combinations of copper-containing inorganic compounds (CuO NPs or Cu salts) as well as Cu–organic complexes with NAC or ascorbate were remarkably cytotoxic for human tumor cell lines, including the variants with the determinants of anticancer drug resistance. Importantly, each component alone evoked little-to-no activity, whereas together, the effect of potentiation was dramatic. Death as a result of the combinations was observed for a variety of cell lines regardless of the tissue origin and was accompanied by rapid (within the initial hours) ROS generation followed by plasma membrane perturbations, such as disappearance of the phosphatidylserine gradient across the membrane (annexin V reactivity) and loss of integrity (PI influx). Cell death was independent of caspase activation or PARP cleavage, strongly suggesting that the plasma membrane (and perhaps other cellular membranes) was the key target of the combinations. These findings are in line with the recently demonstrated efficacy of the combination of Cu (II)-containing compounds and ascorbate in inducing cell cycle perturbation and death via oxidative damage of biomacromolecules [19].

Among a panel of tested metals (Zn, Mo, Fe, and Co), Cu was the only one that evoked cytotoxicity in combination with NAC or cysteine. Phenylalanine, an amino acid that lacks the SH group at the C3 position, was without effect, suggesting the critical role of sulfur. Importantly, the S atom required a specific molecular context, because the S-methyl containing the amino acid methionine did not synergize with CuO. Nevertheless, the S atom was not mandatory since the sulfur-free ascorbate had similar effect to that of NAC, despite being somewhat less pronounced. Altogether, these results point to Cu^2+^ transformation as a mechanism of cell killing.

Electrochemical studies revealed that NAC or ascorbate reduced divalent copper into a monovalent and then into an uncharged state. In cell-free systems, this process depended on the ratio of Cu^2+^ and the reducing agent; 40–100 min were sufficient for the complete transition of the Cu atom’s electric charge to zero. One may hypothesize that reduction took place in the coordination sphere of copper and included the substitution of the SH group for Cl^-^ followed by intramolecular electron transfer from S to Cu atoms and elimination of the S-centered radical from the Cu atom. At the final step, this radical dimerized into disulfide; S atoms became no longer available for copper reduction.

Furthermore, NAC reduced the charge of Cu cations in the context of copper–organic complexes. The presence of the metal was necessary for tumor cell sensitization since NAC showed a weak, if any, effect in combination with Cu-free organic ligands. Importantly, this efficacy significantly differed depending on the molecular environment of the copper ion. As shown in Table 2, the effect of the combination of NAC and compound **1** was much stronger than that of compound **2** + NAC or compound **5** + NAC. This difference can be attributed to the +2 oxidation state of Cu ion in **1** coordinated by monodentate chloride anions in addition to the organic ligand. Complexes **3** and **5** contain copper ions in the oxidation states +1 and +1.5, respectively. Furthermore, in **6** and **8,** copper is tightly coordinated by the tetradentate porphyrin ligand. It can be assumed that, to trigger ROS upon Cu reduction, the coordination compound must fit the following structural requirements: (1) a Cu ion in the complex must be in the +2 oxidation state, and (2) at least one monodentate ligand capable of maintaining the form of a stable anion after reduction must be present.

Electrochemical modifications of Cu ions are mechanistically relevant to the generation of oxidative stress via combinations of inorganic or organic Cu-containing compounds with reducing agents. In the presence of oxygen, Cu^+1^ complexes are capable of generating a superoxide anion that can induce ROS formation in a Fenton-like reaction (Cu^+1^L + O_2_ → O_2_**^−.^** + Cu^+2^L) [30]. We demonstrated intracellular ROS formation within a few hours of exposure to CuO NPs and NAC. In addition to these results, we did not exclude the possibility of the generation of extracellular ROS by these combinations. Indeed, intracellular accumulation of CuO NPs during 24 h cell treatment was as low as ~5% of the initial input as determined by the measurement of copper content in cell lysates by atomic force spectroscopy (S.A.T., V.K.Karandashev and A.A.S., unpublished). Moreover, in the experiments with artificial lipid bilayers, we found no significant change in the membrane permeability caused by CuO NPs in the absence of NAC. The latter agent alone increased the membrane conductance; however, CuO NPs did not augment this effect. We therefore hypothesized that extracellular ROS may represent a major source of oxidative species for cell damage. This suggestion is in line with the molecular ordering of cell death-associated events in which rapid loss of the plasma membrane integrity was the leading factor. Cell-penetrating Cu–organic complexes might be advantageous over CuO NPs in terms of water solubility and intracellular accumulation.

Vulnerability to oxidative stress makes the plasma membrane (as well as membrane organelles) an attractive target for elimination of “intractable” tumor cells otherwise irresponsive to apoptotic stimuli [31,32]. We found that CuO NPs + NAC efficiently killed the K562/4 (P-glycoprotein mediated MDR), SCOV-3/CDDP (resistance to cisplatin; [24] and HCT116p53KO (altered response to DNA damaging agents; [25] sublines; the potency of the combination was similar among these sublines and their parental counterparts. However, non-malignant fibroblasts were sensitive to the combination of CuO NPs and NAC, indicating no cell-type selectivity of the combination.

Nevertheless, the combinations analyzed herein can be applied to advanced disease where conventional treatments usually fail, e.g., for the purge of thoracic or peritoneal cavities from metastatic cells. In these situations, the efficacy of therapies aimed at induction of apoptosis is unlikely; instead, salvage treatment strategies should be considered. The escape of metastatic cells cannot be solely attributed to an impairment of individual death signaling pathways. Mechanisms such as an altered intracellular metal transport can also be advantageous for tumor cell survival. In this scenario, Cu reduction and ROS generation in the extracellular milieu are perspective for potent and irreversible damage of metastatic cells.

Recently, Wu et al. [33] reported a tumor-specific “non-toxicity-to-toxicity” transition strategy based on hollow mesoporous silica NPs as the carrier. The particles doped with Cu^2+^ and the FDA-approved drug disulfiram released these components under acidic conditions of the tumor microenvironment. The in situ chelation reaction between the co-released Cu^2+^ ions and disulfiram generated toxic products. Concurrently, Fenton-like reactions between the generated Cu^+^ ions and H_2_O_2_ resulted in ROS production. This strategy was efficacious against tumor xenografts. Thus, biocompatible materials for targeting tumors with locally controllable cytocidal oxidative burst provide a valuable approach to eradicating intractable malignancies.

In the present study, we used undecorated CuO NPs, proving their efficacy. Future developments should address the targeted delivery of Cu-containing materials using surface coating with antibodies to identify tumor-specific antigens. Furthermore, given the ability of sulfur to reduce copper in different molecular contexts, the design of supramolecular systems to guide Cu^2+^ to critical cysteine residues for oxidative destruction of targeted proteins has emerged as a practically important problem.

## 4. Materials and Methods

### 4.1. Metal (II)-Containing Agents 

Iron chloride hexahydrate (FeCl_3_∙6H_2_O), iron (II) sulfate (FeSO_4_∙7H_2_O), and cobalt nitrate hexahydrate (Co(NO_3_)_2_∙6H_2_O), as well as other reagents unless specified otherwise, were purchased from Sigma-Aldrich. CuO NPs were synthesized using the precipitation method [21]. Briefly, 0.5 mg of CuSO_4_∙5H_2_O (Reachim, Saint-Petersburg, Russia) was dissolved in 100 mL of deionized water to obtain a 10 mM solution, which was heated at 98 °C. Sodium hydroxide (1 M; LenReactiv, Saint-Petersburg, Russia) was added to the mixture under vigorous stirring. The instantly formed black precipitate was washed twice with ethanol and twice with deionized water by pelleting at 12,000 g for 10 min. The size of the formed NPs was determined by dynamic light scattering (DLS) using a Photocor EPM/Photocor Compact-Z analyzer (Photocor, Moscow, Russia). ZnO NPs were synthesized using the same procedure as that for CuO NPs. A solution of 1.5 g Zn(NO_3_)_2_∙6H_2_O in 60 mL water was heated to 91 °C followed by the addition of 1 mL 1 M NaOH. Formation of white color indicated the completion of the reaction. Magnetite (Fe_3_O_4_) NPs were synthesized as described [34]. In addition, divalent metal salts Co(NO_3_)2_*_6H_2_O and MoS_2_ (Alfa Aesar, Ward Hill, MA, USA) were tested. Materials were stored at 4 °C as dry powders.

### 4.2. Cu (II)-Containing Organic Complexes

Synthesis of Cu-containing organic complexes [(Z)-3-(2-fluorophenyl)-2-methylthio-5-(pyridin-2-ylmethylene)-3,5-dihydro-4H-imidazol-4-one]copper(II) dichloride (**1**; Scheme 1), [(Z)-3-(2-fluorophenyl)-2-methylthio-5-(pyridin-2-ylmethylene)-3,5-dihydro-4H-imidazol-4-one]copper(I) chloride (**3**) and bis[(Z)-3-(2-fluorophenyl)-2-thio-5-(pyridin-2-ylmethylene)-3,5-dihydro-4H-imidazol-4-one]copper(+1.5) µ-chloride (**5**), and corresponding organic ligands [(Z)-3-(2-fluorophenyl)-2-methylthio-5-(pyridin-2-ylmethylene)-3,5-dihydro-4H-imidazol-4-one (**2**) and (Z)-3-(2-fluorophenyl)-2-thio-5-(pyridin-2-ylmethylene)-3,5-dihydro-4H-imidazol-4-one (**4**) has been reported elsewhere [10].

Synthesis of the Cu complex of 2-(3′-maleimido)-5,10,15,20-tetraphenylporphyrin **6** and the metal free 2-(3′-maleimido)-5,10,15,20-tetraphenylporphyrin **7** has been reported in [24]. The copper complex of 5-[*p*-(3′-maleimido)phenyl]-10,15,20-triphenylporphyrin; compound **8**) was synthesized as follows: a solution of 50 mg (0.25 mmol) Cu(OAc)_2_·H_2_O in methanol (5 mL) was added to a solution 50 mg (0.07 mmol) 5-[*p*-(3′-maleimido)phenyl]-10,15,20-triphenylporphyrin **9** in methylene chloride (5 mL). The resulting mixture was stirred for 1.5 h at room temperature with TLC monitoring (CHCl_3_–hexane 1:1). Then, the reaction mixture was poured into water and extracted with methylene chloride. The organic layer was dried over Na_2_SO_4_, and the solvent was removed under reduced pressure. Yield 50 mg (93%, dark red solid). UV–Vis (CH_2_Cl_2_) λ_max_, (ε × 10^−3^) nm: 419 (253), 541 (10.7), 577 (2.4). IR (KBr) ν_max_, cm^−1^: 1719 (C=O), 1597 (C=C). The metal-free 5-(*p*-(3′-maleimido)phenyl)-10,15,20-triphenylporphyrin (compound **9**) was previously synthesized by us [29]. Chemical structures of compounds **1**–**9** are shown in Scheme 1.

### 4.3. Cell Lines and Treatment

The panel of tumor cell lines included human K562, KU-812, and MOLM-6 chronic myelogenous leukemia; HCT116 colon, SKOV-3 ovarian, MCF-7, and MDA-MB-231 breast carcinomas; and B16F10 murine melanoma (all from American Type Culture Collection (Manassas, VA, USA)). In addition, the isogenic sublines with the molecular determinants of altered drug response were multidrug resistant *MDR*1/P-glycoprotein overexpressing K562/4 subline, HCT116p53KO (non-functional p53) and SKOV-3/CDDP (a 4-fold resistance to cisplatin; gift of G.A.Posypanova, National Research Center ‘The Kurchatov Institute’, Moscow, Russia) [24,25,35]. Non-malignant hFB-hTERT6 skin fibroblasts were obtained via a lentiviral transduction of the full-length *TERT* gene under a cytomegalovirus promoter (gift of E. Dashinimaev, Engelhardt Institute of Molecular Biology, Moscow). Cells were maintained in RPMI-1640 (K562, K562/4, KU-812, and MOLM-6) or Dulbecco mediated Eagle’s medium (other cell lines) supplemented with 10% fetal bovine serum (HyClone, Logan, UT, USA) and 50 μg/mL gentamicin at 37 °C, 5% CO_2_ in a humidified atmosphere. The freshly prepared aqueous suspensions of inorganic materials (see above) were added to the cell-free systems or cell cultures. Copper organic complexes and the respective metal-free organic ligands were dissolved in dimethyl sulfoxide (10 mM). Aqueous solutions were prepared on the day of experiments. N-acetylcysteine (NAC) and cysteine were dissolved in culture media at 50 mM stock solutions, and then pH was adjusted to 7.2–7.4 with 1 M NaOH. Stock solutions of sodium ascorbate (500 mM) and α-tocopherol (250 mM) were prepared in water or used as oil, respectively. The pan-caspase inhibitor N-benzyloxycarbonyl-Val-Ala-Asp(O-Me) fluoromethyl ketone (z-VAD-FMK) was from Selleckchem, Houston, TX, USA. The cytotoxicity of metal NPs and Cu–organic complexes alone or in combinations with NAC, ascorbate, or α-tocopherol was determined in MTT assays [36].

### 4.4. Cell Fluorescence

Cell associated fluorescence was recorded on a CytoFlex flow cytometer (Beckman Coulter, Brea, CA, USA). At least 10,000 events were collected per sample. Data were analyzed using CytExpert software (Beckman Coulter).

### 4.5. Time Course of Cell Death

CuO NPs (0.1 µg/mL) and NAC (1 mM) were added to the K562 cells in 24-well plates (5 × 10^4^ per well in 1 mL medium). Every hour, cell aliquots were collected and incubated with propidium iodide (PI; 10 µg/mL) for 2 min in the dark. The intensity of fluorescence was measured in the phycoerythrin (PE) channel (585/42 nm).

### 4.6. Annexin V-FITC/PI Staining

K562 cells were plated into 6-well plates (Eppendorf; 2 × 10^5^ cells/well) and treated with the combination of CuO NPs (0.1 µg/mL) and NAC (1 mM) for 4–24 h. Then, cells were stained with annexin V-FITC for 15 min at room temperature, washed with PBS, and counterstained with 10 µg/mL PI (Dead Cell Apoptosis Kit for flow cytometry; Thermo Fisher Sci., Waltham, MA, USA). Fluorescence intensity was detected in FITC (525/40 nm) and PE channels.

### 4.7. Cell Cycle Distribution

K562 cells (2 × 10^5^) were plated into 6-well plates overnight and then treated with 0.1 µg/mL CuO NPs and 1 mM NAC for 6 h or 24 h. Cells were pelleted and lysed in the buffer containing 50 µg/mL PI, 0.1% sodium citrate, 100 µg/mL RNase A, and 0.3% NP-40 for 30 min in the dark. Fluorescence was detected in the PE channel.

### 4.8. Mitochondrial Transmembrane Potential

The MitoTracker™ Red CMXRos (Thermo Fisher Sci.) was used to monitor the mitochondrial potential. K562 cells were treated with a combination of CuO NPs (0.1 µg/mL) and 1 mM NAC for 4–24 h. The MitoTracker (final concentration 85 nM) was added to the cells 30 min prior to the completion of treatment. Then, the cells were washed with cold PBS and analyzed in the allophycocyanin (APC) channel (660/10 nm).

### 4.9. ROS Detection

The 6-carboxy-2′,7′-dichlorodihydrofluorescein diacetate (carboxy-H_2_DCFDA) dye (Thermo Fisher Sci.) was used to detect intracellular ROS. K562 cells (2 × 10^5^ in 1 mL of medium) were treated with 5 µM carboxy-H_2_DCFDA for 30 min, washed, and resuspended in fresh medium. CuO (1 µg/mL), NAC (1 mM), or their combination was added to corresponding tubes, and cells were incubated at 37 °C, 5% CO_2_ for 1–4 h. Cell associated fluorescence of carboxy-H_2_DCFDA was measured by flow cytometry in the FITC channel. Ten thousand events were collected per sample. Values of mean fluorescence intensities were normalized by the respective values in untreated (DCFDA alone) cells.

Laser scanning confocal microscopy was used to visualize the morphology of mitochondria and to monitor the integrity of the plasma membrane upon treatment with CuO NPs and NAC. The HCT116 cells were plated on 35 mm dishes with the glass bottom (SPL Life Sci., Gyeonggi-do, Korea) and incubated for 72 h at 37 °C, 5% CO_2_ to reach 50% confluence. Then, the cells were incubated with CuO NPs (1 µg/mL) and NAC (1 mM) for 1–24 h followed by washing with PBS. Photolon (10 µM, 3 h, illumination at 660 nm, 33 J/cm^2^) was a reference compound for light-activated oxidative stress and rapid organelle damage, a property of chlorin e6 derivatives [26,37]. For labeling mitochondria and nuclei, dihydrorhodamine 123 (DHR 123; (488 nm/510–570 nm)) and Hoechst 33342 (405 nm/415–470 nm), respectively, were used as recommended by the manufacturer (Thermo Fisher Sci.). The entry of PI (488 nm/550–600 nm) was used to monitor the plasma membrane integrity. Images were analyzed on a Leica TCS SPE 5 laser scanning confocal microscope with LAS AF software (Leica Microsystems GmbH, Wetzlar, Germany).

### 4.10. Immunostaining

K562 cells were treated with 0.1 µg/mL CuO NPs in the absence or presence of 1 mM NAC for 6 h and 24 h. After fixation in 0.75% paraformaldehyde for 15 min and permeabilization in ice cold methanol for 30 min, cells were washed with PBS and incubated with rabbit antibodies against cleaved caspase 3 and PARP (Cell Signaling, Danvers, MA, USA; 1:1000) for 1 h. After washing with PBS, the secondary goat anti-rabbit antibodies conjugated with AlexaFluor 488 (Thermo Fisher Sci.; 1:1000) were added, and cells were incubated for 1 h and analyzed by flow cytometry in the FITC channel.

### 4.11. Immunoblotting

The K562 and HCT116 cells in 60 mm Petri dishes (80% confluency) were treated with CuO NPs (0.1 µg/mL) and NAC (1 mM) for 24 h at 37 °C, 5% CO_2_. Dox (0.2 µM) was used as a reference compound. After the completion of treatment, the cells were lysed in the buffer containing 150 mM NaCl, 1% NP-40, 0.1% SDS, 50 mM Tris base pH 8.0, 2 mM phenylmethylsulfonyl fluoride, and protein inhibitor cocktail (Roche) for 30 min on ice. The protein concentration was evaluated using Bradford reagent. Cell lysates (35 µg of total protein) were resolved in 12% sodium dodecyl sulfate-polyacrylamide gel (SDS-PAGE) at 120 V for 1.5 h and transferred onto 0.2 µm nitrocellulose membranes (GE Healthcare, Chicago, IL, USA). After blocking with skimmed milk, the membranes were incubated with rabbit antibodies against human cleaved PARP and total or cleaved caspase 3 (Cell Signaling) at 4 °C overnight, washed, and incubated with secondary goat anti-rabbit antibodies conjugated with horseradish peroxidase (Cell Signaling). Proteins were visualized using the enhanced chemiluminescence reagent and a ChemiDoc MP gel imaging system (BioRad, Hercules, CA, USA).

### 4.12. Planar Lipid Bilayer Setup, Recording System, and Mode of Calculations

Synthetic 1-palmitoyl-2-oleyl-*sn*-glycero-3-phosphocholine (POPC) and1-palmitoyl-2-oleyl-*sn*-glycero-3-phospho-(1′-glycerol) (POPG) were obtained from Avanti Polar Lipids, Inc. (Pelham, AL, USA). KCl solution (0.1 M) was buffered with 5 mM HEPES, pH 7.4. Bilayer lipid membranes were prepared from POPC or POPG using a monolayer-opposition technique [38] on a 50-µm-diameter aperture in a 10-µm-thick Teflon film separating *cis*- and *trans*-compartments of the Teflon chamber. Experiments were performed in the chambers containing 0.1 M KCl, 5 mM HEPES-KOH pH 7.4. NAC (1 mM) and CuO NPs (1–4 µg/mL) (each agent alone or in combinations) were added to the *cis*-chamber to mimic the physiologically relevant conditions. Ag/AgCl electrodes with 1.5% agarose/2 M KCl bridges were used to apply the transmembrane voltage (*V*) and measure the transmembrane current (*I*). “Positive voltage” refers to the case in which the *cis*-side compartment is positive with respect to the *trans*-side. All experiments were performed at room temperature.

Measurements of electric current were carried out using an Axopatch 200B amplifier (AutoMate Scientific Inc., Berkeley, CA, USA) in the voltage-clamp mode. Data acquisition was performed with a 5 kHz sampling frequency and low-pass filtering at 100 Hz. The current tracks were processed through an 8-pole Bessel 100 kHz filter. Data were digitized by Digidata 1440A and analyzed using pClamp 10 (AutoMate Sci.) and Origin 8.0 (OriginLab Corp., Northampton, MA, USA).

### 4.13. Calcein Release from Liposomes

Large unilamellar vesicles were made from POPC or POPG and loaded with the fluorescent dye calcein (35 mM) using a mini-extruder (Avanti Polar Lipids, Alabaster, AL, USA). At this concentration, calcein fluorescence inside the liposomes is self-quenched. The increase in fluorescence of free calcein in the surrounding media is a measure of the disturbance of membrane integrity in the absence and presence of NAC (1 mM), CuO NPs (1–4 µg/mL), or their combinations. The fluorescence of released calcein was determined on a Fluorat-02-Panorama spectrofluorometer (Lumex, Saint-Petersburg, Russia) at λ_ex_ = 490 nm, λ_em_ = 520 nm. The detergent Triton X-100 (at final concentration of 1%) was added at the end of experiments for complete disruption of liposomes (referred to full disengagement of the marker from vesicles).

The relative intensity of calcein fluorescence (*IF*, %) was calculated as follows:

(1) $$IF=\frac{I-I_{0}}{I_{\max}/0.9-I_{0}}\cdot 100\%$$

where *I* and *I*_0_ are the calcein fluorescence intensities in the presence and absence of NAC (1 mM), CuO NPs (1–4 µg/mL), or their combinations, respectively, and *I_max_* is the maximal fluorescence after treatment of liposomes with Triton X-100. A factor of 0.9 was introduced to account for sample dilution by Triton X-100.

### 4.14. Differential Scanning Microcalorimetry (DSC) of Liposomal Suspensions

Experiments were performed on a μDSC 7 EVO microcalorimeter (Setaram, France). Giant unilamellar vesicles were formed from 1,2-dipalmitoyl-*sn*-glycero-3-phosphocholine (DPPC) or 1,2-dipalmitoyl-*sn*-glycero-3-phospho-1′-glycerol (DPPG) (Avanti Polar Lipids, Inc. (Pelham, AL, USA) by the electroformation method (standard protocol, 3V, 10 Hz, 1 h, 55 °C) using the Nanion vesicle *prep pro* device (Nanion Technologies, Munich, Germany). Final concentrations were 3 mM, 1 mM, and 1–4 µg/mL of DPPC/DPPG, NAC and CuO NPs, respectively. The liposomal suspension was heated at a constant rate of 0.2 C/min. Reversibility of thermal transitions was assessed by reheating the sample immediately after the cooling step from the previous scan. The temperature dependence of the excess heat capacity was analyzed using Calisto Processing (Setaram, Caluire-et-Cuire, France). The thermograms were characterized by temperature of the main phase transition of DPPC or DPPG (*T_m_*), and the half-width of main peak (*T*_1/2_) that characterizes the size of the cooperative lipid unit.

### 4.15. Electrochemical Measurements of Cu Redox State

The electrochemical behavior of CuO NPs and compounds **1–5** was studied using cyclic voltammetry (CV) and rotating disk electrode (RDE) techniques. An IPC Pro M potentiostat was used for electrochemical studies. The working electrode was a glassy carbon disk (d = 2 mm), and the reference electrode was Ag/AgCl/KCl (sat.). The auxiliary electrode was a platinum plate, and the supporting electrolyte was 0.1 M Bu_4_NClO_4_ solution in dimethylformamide. The potential sweep rates were 100 mV/s (CV method) and 20 mV/s (RDE method). Samples were dissolved in an air-free solvent. All measurements were carried out in a dry argon atmosphere.

### 4.16. Mass Spectrometry

For HPLC analysis, a system with a Shimadzu Prominence LC-20 (Shimadzu Corporation, Kioto, Japan) column and a convection fraction collector connected with a single quadrupole mass spectrometer Shimadzu LCMS-2020 (Shimadzu Corporation, Kioto, Japan) with dual ionization source DUIS-ESI-APCI were used. The analytical and preparative column was Phenomenex Luna 3u C18 100A.

### 4.17. Measurement of Thiol Group Content

The 5,5-dithiobis(2-nitrobenzoic acid) (DTNB; Ellman’s reagent) (Thermo Fisher Sci.) was used to determine the sulfhydryl groups. The solution of 2 mM DTNB in 50 mM sodium acetate was mixed with 1 M Tris base pH 8.0 (1:2 *v*/*v*) to obtain the working solution. The reaction mixture (400 µL) contained 100 ng/mL or 1 µg/mL CuO NPs alone, 1 mM NAC alone or the combinations of each concentration of NPs with NAC. An aliquot (10 µL) was immediately transferred into a 96-well plate (Eppendorf), and then 190 µL of the working solution was added. Values of optical density (OD) at 412 nm were measured on a Tecan Spark 10M spectrophotometer (Tecan, Mennedorf, Switzerland).

### 4.18. Statistical Analysis

Experiments were carried out in 3–4 replicates. Data are presented as mean ± SD. Statistical analysis was performed using GraphPad Prism 7 and Microsoft Office Excel software.

## Figures and Tables

**Table 1:** NAC significantly enhances the cytotoxicity of CuO NPs.

Cell Line	CuO *	CuO + 1 mM NAC *	Fold Sensitization **
K562	8.5	0.01	850
K562/4	16.2	0.01	1620
MDA-MB-231	18.3	0.06	305
HCT116	26.8	0.05	536
HCT116p53КО	15.2	0.04	380
MOLM-6	5.7	0.02	285
KU-812	7.0	0.02	350
B16F10	17.6	0.06	293
MCF-7	45.2	0.05	904
SCOV-3	3.9	0.09	42
SCOV-3/CDDP	4.3	0.37	12
hFB-hTERT6	9.1	0.02	455

* IC_50_ (µg/mL), mean of 3 experiments with <10% error. ** ratio IC_50CuO_/IC_50CuO+NAC_.

**Table 2:** Sensitization of HCT116 cells to Cu–organic compounds by NAC.

Compound	No NAC *	+ 1 mM NAC *	Fold Sensitization **
**1**	109.2	0.3	364.0
**3**	6.9	0.5	13.8
**4**	6.3	3.1	2.0
**5**	4.2	0.6	7.0
**6**	759.0	37.4	20.3
**8**	474.5	44.4	10.7

* IC_50_ (µg/mL), mean of 3 experiments with <10% error. ** IC_noNAC_/IC_compound+NAC_ ratio.

## Data Availability

Not applicable.

## Supplementary Materials

The following are available online at https://www.mdpi.com/article/10.3390/ijms222011065/s1.
