# Supplementary material for: Copper-Containing Nanoparticles and Organic Complexes: Metal Reduction Triggers Rapid Cell Death via Oxidative Burst
Source: Int J Mol Sci. 2021 Oct 14;22(20):11065. doi: 10.3390/ijms222011065 (PMC8539714; doi:10.3390/ijms222011065)
Supplement: Supplementary file 1 [file ijms-22-11065-s001.zip › ijms-1397404-supplementary.pdf]

Copper-Containing Nanoparticles and Organic Complexes: Metal Reduction Triggers Rapid Cell Death via Oxidative Burst

Sergey A. Tsymbal [1,*], Anna A. Moiseeva [2], Nikol A. Agadzhanian [1], Svetlana S. Efimova [3], Alina A. Markova [4,5], Dmitry A. Guk [2], Olga O. Krasnovskaya [2], Victoria M. Alpatova [5], Andrei V. Zaitsev [5], Anna V. Shibaeva [4], Victor V. Tatarskiy [6], Marina S. Dukhinova [1], Valentina A. Ol'shevskaya [5], Olga S. Ostroumova [3], Elena K. Beloglazkina [2,†] and Alexander A. Shtil [2,7,†]

[1] International Institute of Solution Chemistry of Advanced Materials and Technologies, ITMO University, 197101 Saint-Petersburg, Russia; agadzhanian@scamt-itmo.ru (N.A.A.); dukhinova@scamt-itmo.ru (M.S.D.)
[2] Department of Chemistry, Moscow State University, 119991 Moscow, Russia; moiseeva@org.chem.msu.ru (A.A.M.); dmh200949@gmail.com (D.A.G.); krasnovskayao@gmail.com (O.O.K.); beloglazki@mail.ru (E.K.B.); shtilaa@yahoo.com (A.A.S.)
[3] Institute of Cytology, Russian Academy of Sciences, 194064 Saint-Petersburg, Russia; efimova@incras.ru (S.S.E.); osostroumova@mail.ru (O.S.O.)
[4] Emanuel Institute of Biochemical Physics, Russian Academy of Sciences, 119334 Moscow, Russia;
alenmark25@gmail.com (A.A.M.); anna-shiba@mail.ru (A.V.S.)
[5] A.N.Nesmeyanov Institute of Organoelement Compounds, Russian Academy of Sciences, 119334 Moscow, Russia; vika.alpatova@gmail.com (V.M.A.); porphyrin@yandex.ru (A.V.Z.); olshevsk@ineos.ac.ru (V.A.O.)
[6] Institute of Gene Biology, Russian Academy of Sciences, 119334 Moscow, Russia; tatarskii@gmail.com
[7] Blokhin National Medical Research Center of Oncology, 115478 Moscow, Russia; shtilaa@yahoo.com (A.A.S.)
* Correspondence: Correspondence: zimbal@scamt-itmo.ru
† These authors share senior authorship.

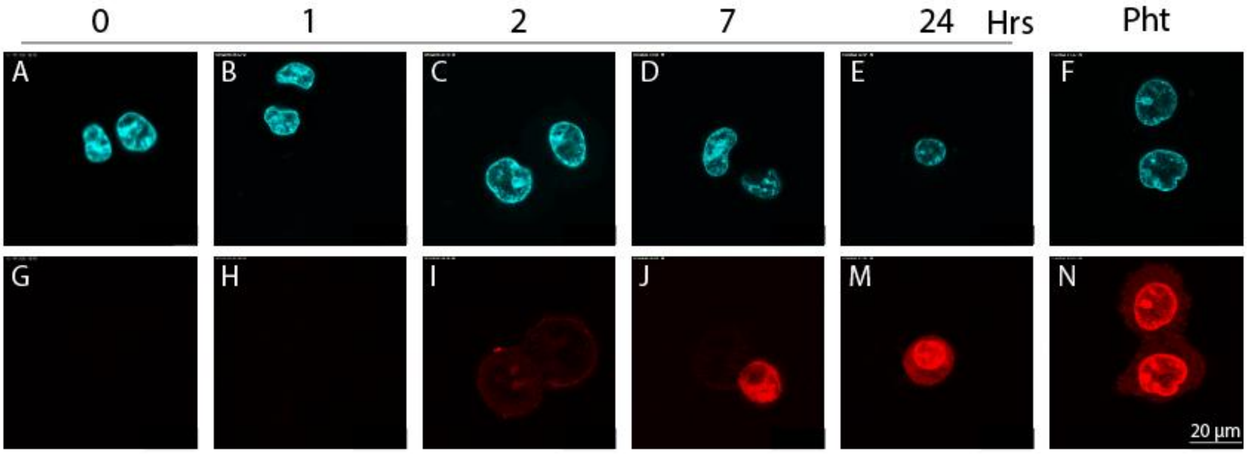

Figure S1. Time course of the plasma membrane damage by CuO+NAC. The HCT116 cells were treated with 1 µg/ml CuO+1 mM NAC for indicated time intervals or with photolon (Pht; 10 µM, 3 h) and stained with Hoechst 33342 (*top panel*) or PI (*bottom panel*). Images were taken on a Leica TCS SPE laser confocal microscope. Shown is one representative experiment out of 3 with similar results.

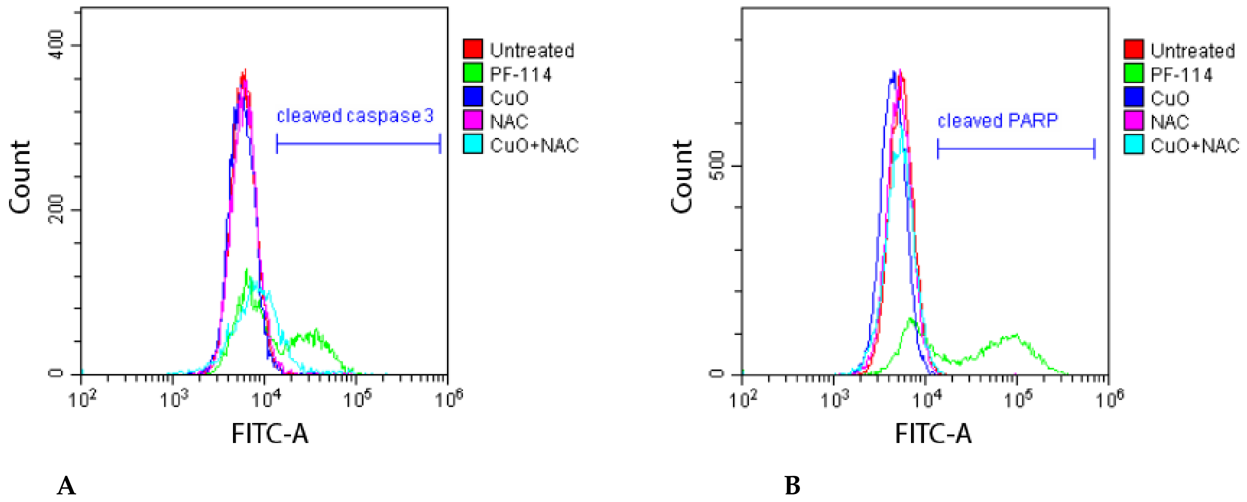

Figure S2. Lack of caspase 3 activation and PARP cleavage in K562 cells treated with CuO+NAC. . Immunostaining of K562 cells for cleaved caspase 3 (A) and PARP (B). PF-114, a Bcr-Abl inhibitor, is used as a reference inducer of apoptosis in Bcr-Abl positive K562 cells (see main text for details).

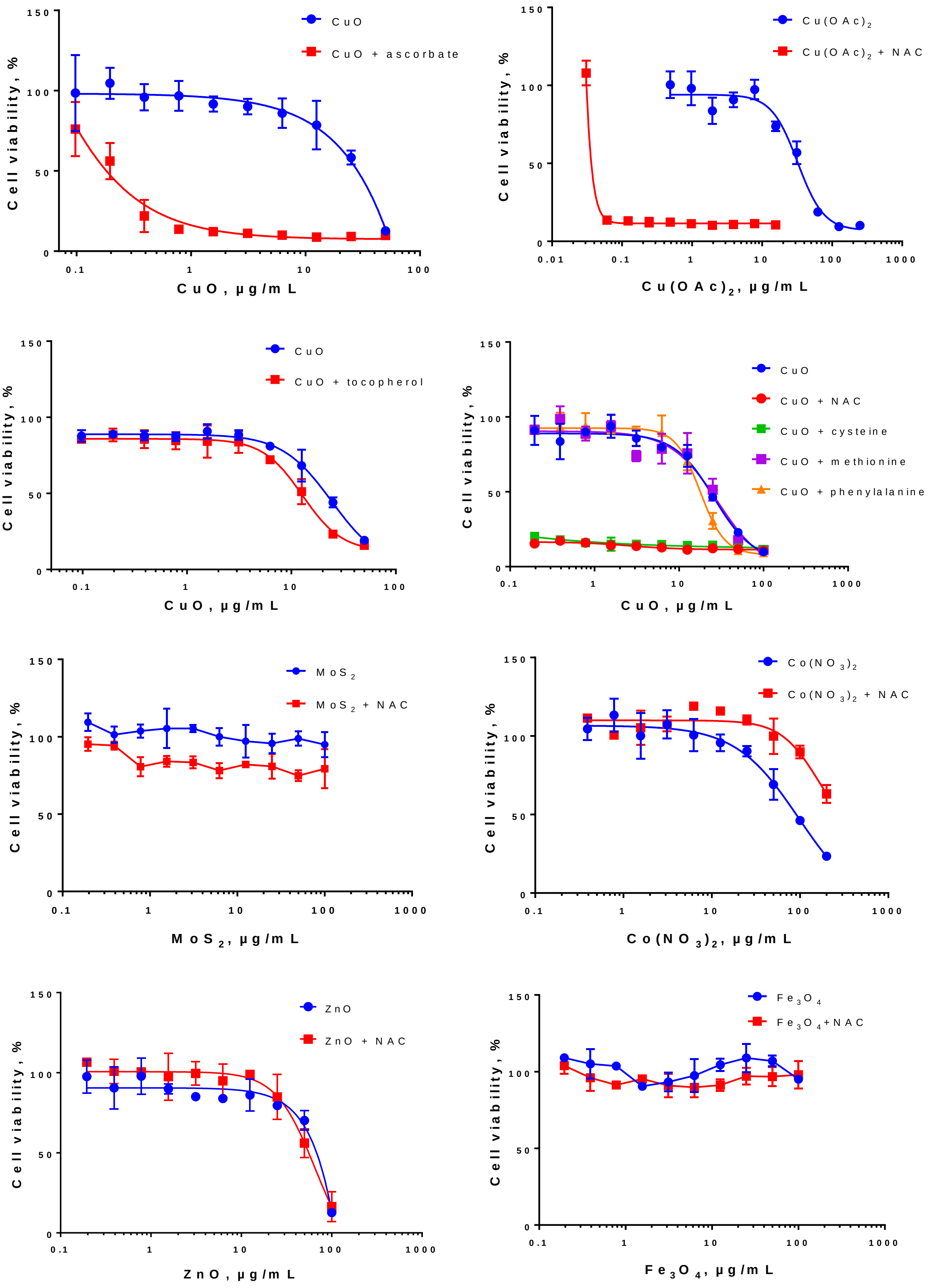

Figure S3. Differential efficacy of combinations of metal containing compounds with ascorbate, α-tocopherol and amino acids. Shown are the results of MTT tests, each value is mean ± S.D. of 3-4 independent measurements. NAC, 1 mM.

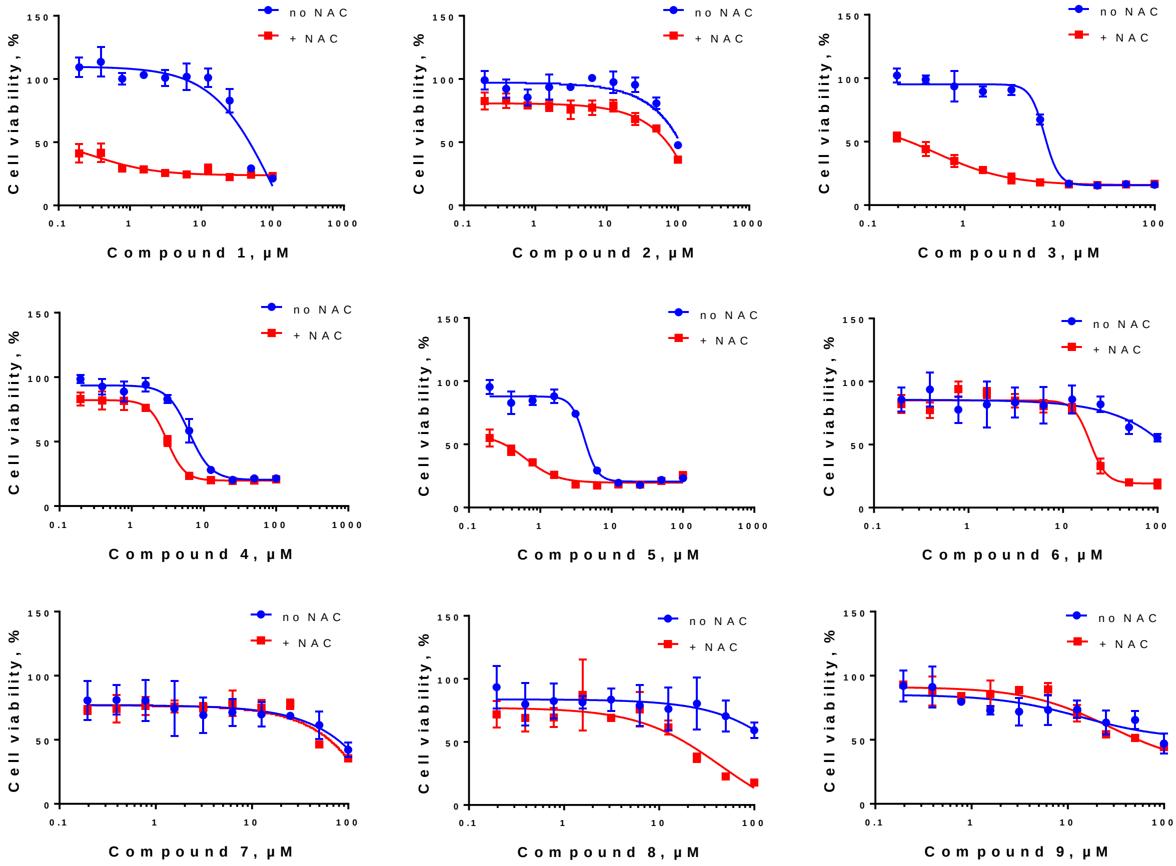

Figure S4. Cytotoxicity of Cu-organic compounds and metal free carcasses for HCT116 cells. NAC, 1 mM. Shown are the results of MTT tests, each value is mean ± S.D. of 3-4 independent measurements.

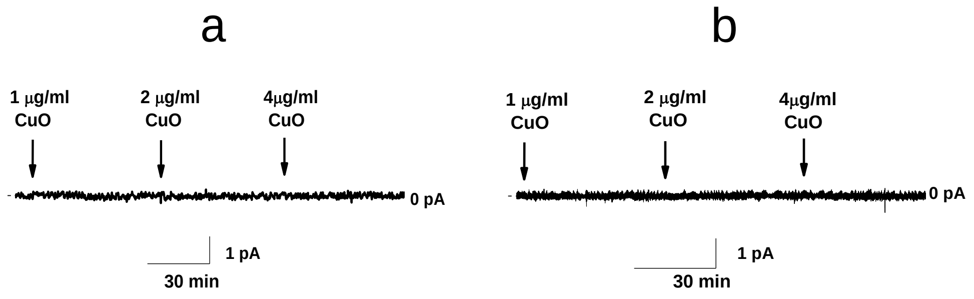

Figure S5. Current fluctuations induced by CuO NPs (1-4 μg/ml) in lipid bilayers composed of POPC (**a**) and POPG (**b**) and bathed in 0.1 M KCl pH 7.4. The transmembrane voltage was 50 mV. Arrows indicate the addition of CuO NPs to the membrane bathing solution.

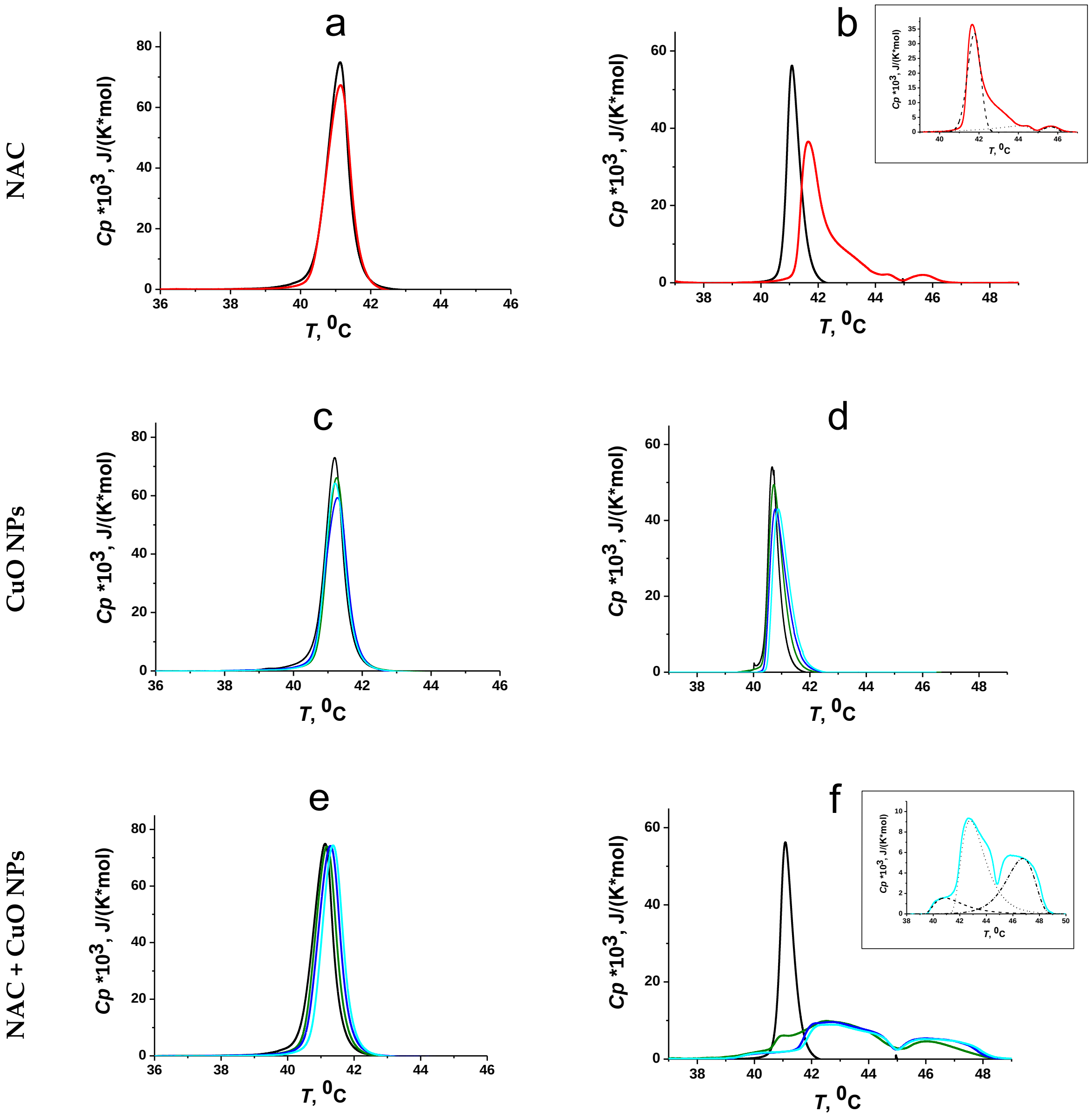

Figure S6. Heating DSC thermograms of DPPC (a,c,e) and DPPG (b,d,f) liposomes in the absence (*black lines*) and presence of NAC (1 mM) (a,b, *red lines*), CuO NPs (c,d) (1 µg/ml, *green lines*; 2 µg/ml, *blue lines*; 4 µg/ml, *cyan lines*) or in combination (e,f). Shown are the results of one representative scan out of two independent measurements and two cool-heat scan steps for each system.

LC-MS data

Chromatogram

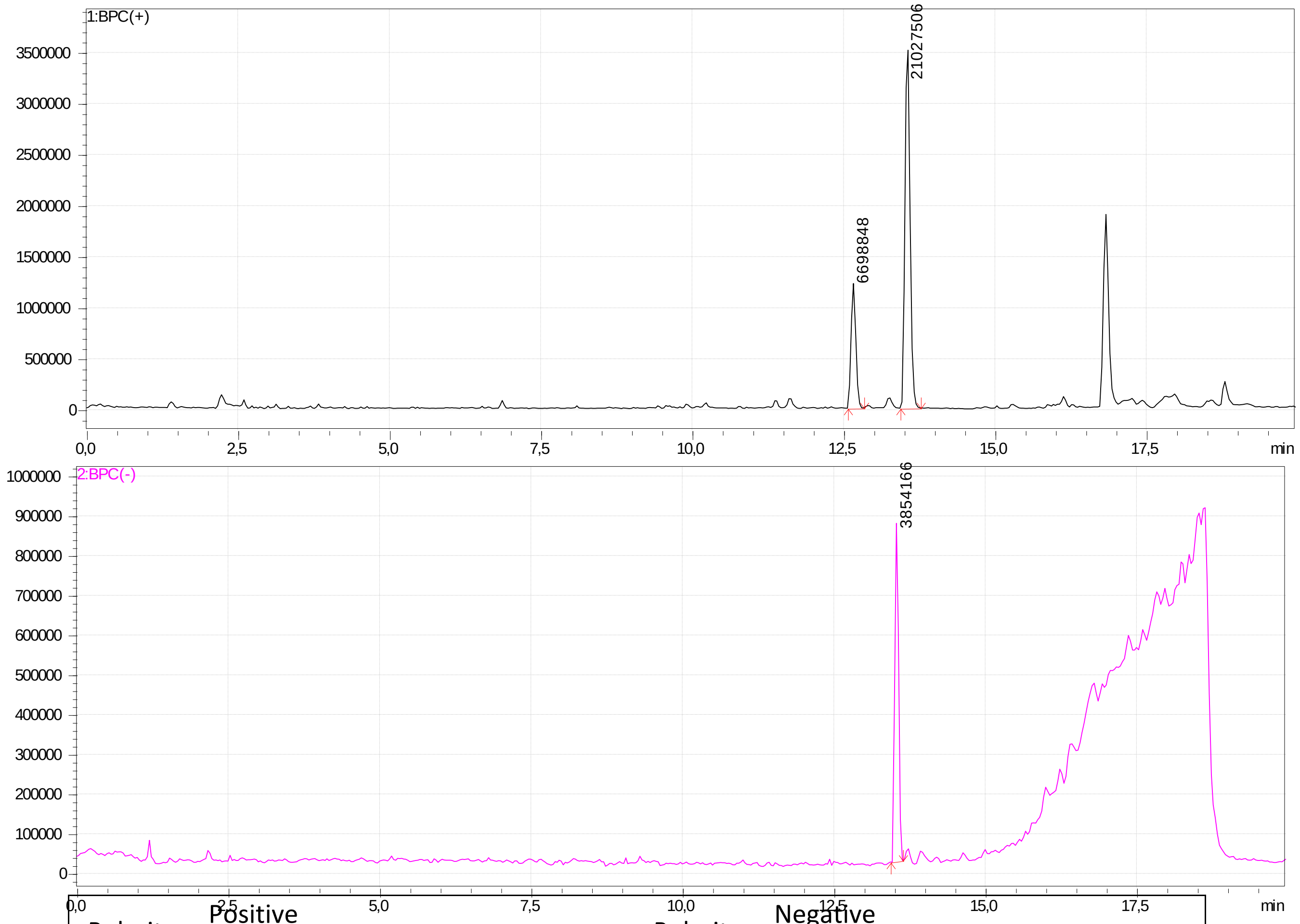

Polarity	Positive ion			Polarity	Negative ion		
Peak#	Ret. Time	Base Peak m/z	Area%	Peak#	Ret. Time	Base Peak m/z	Area%
1	12,664	314,05	24,161	1	13,538	282,1	100
2	13,556	284,1	75,839				

Spectra

Polarity Positive ion

Peak# 1

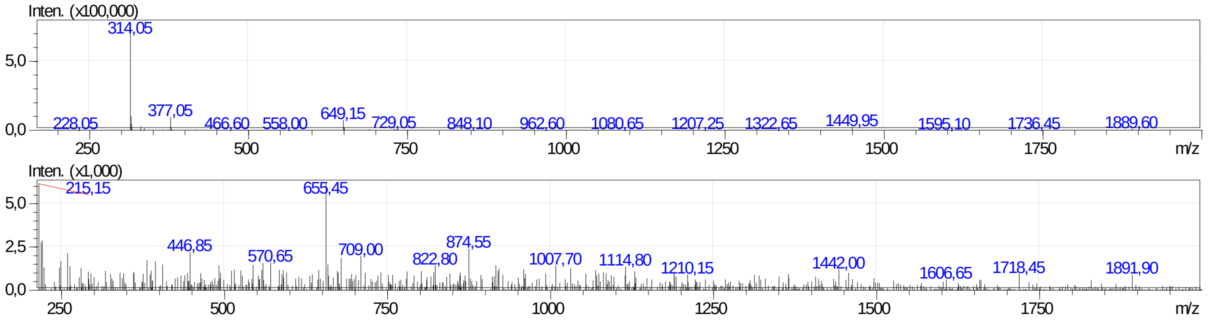

Peak# 2

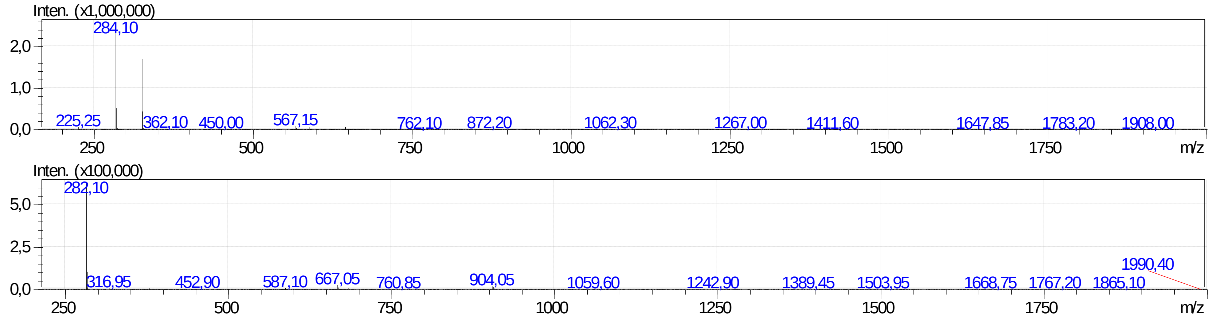

Polarity Negative ion

Peak# 1

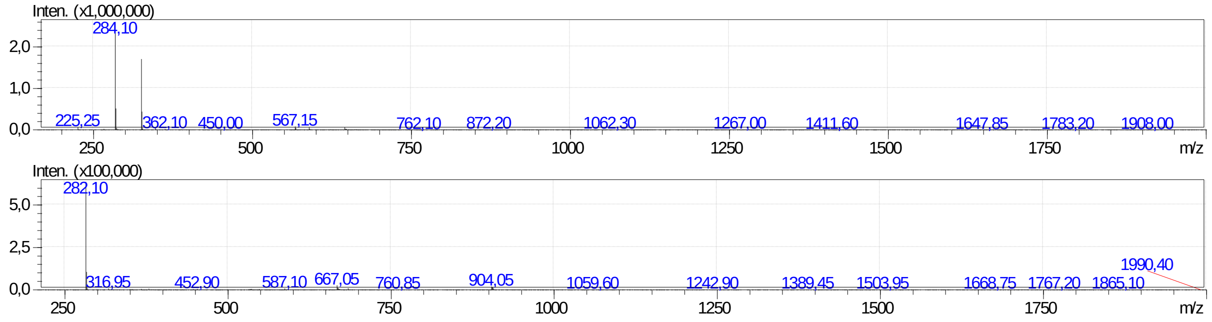

Figure S7. LC-MS mass spectra of the supernatant obtained by the reaction of **1** with NAC.

Chromatogram

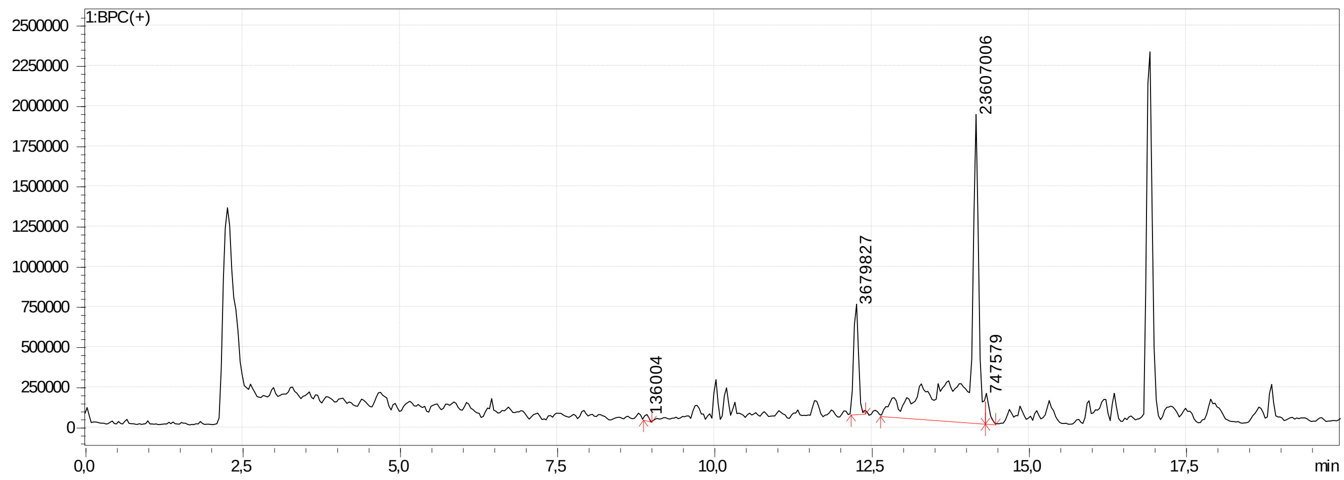

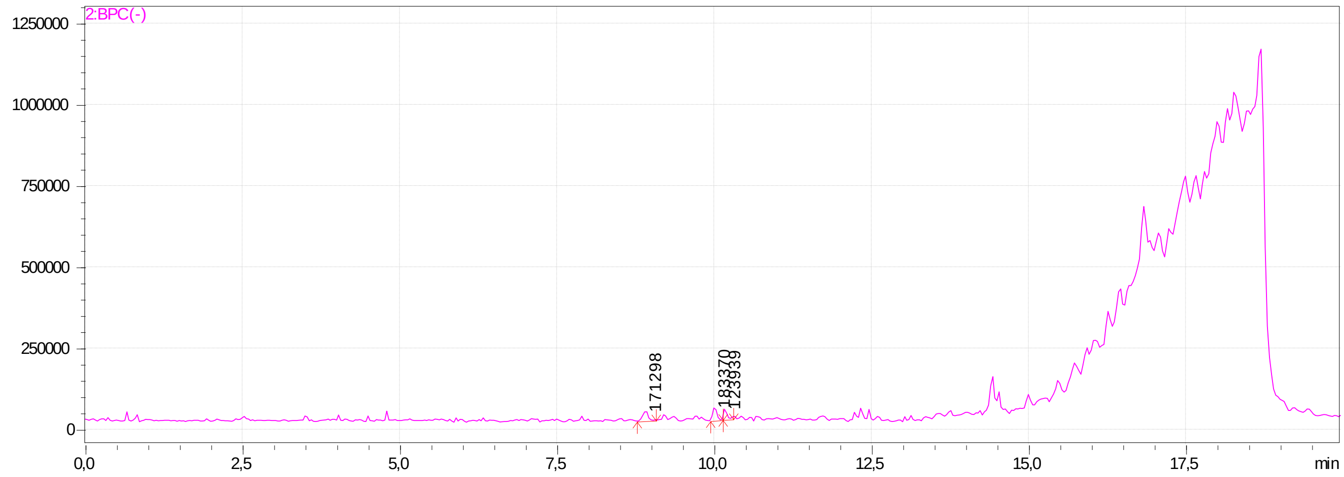

Polarity	Positive ion			Polarity	Negative ion		
Peak#	Ret. Time	Base Peak m/z	Area%	Peak#	Ret. Time	Base Peak m/z	Area%
1	8,926	325,05	0,483	1	8,917	322,95	35,791
2	12,26	286	13,063	2	10,011	514,3	38,313
3	14,166	272,1	83,801	3	10,174	627,4	25,896
4	14,33	437,2	2,654				

Polarity Positive ion

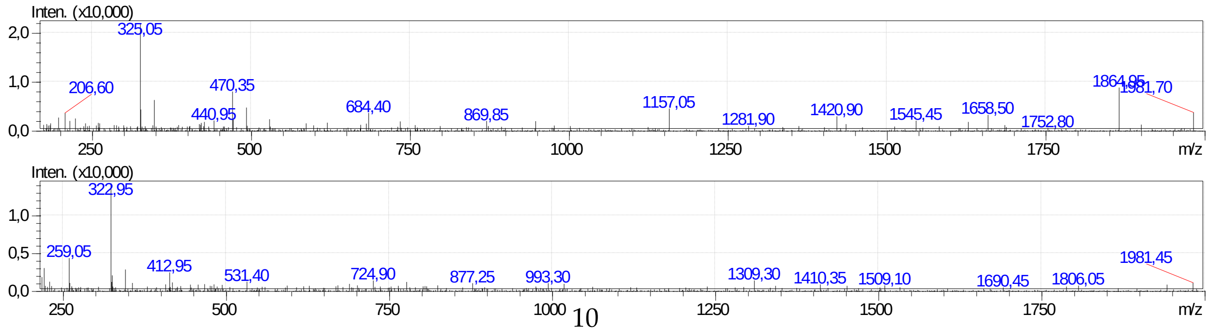

Peak# 2

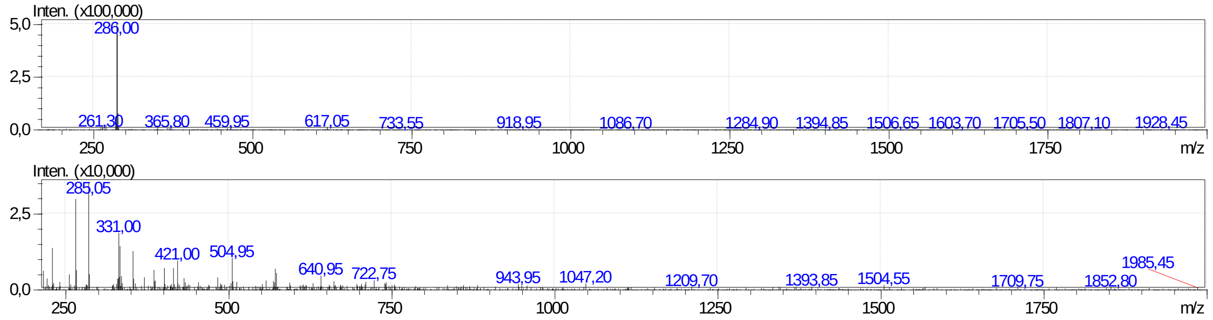

Peak# 3

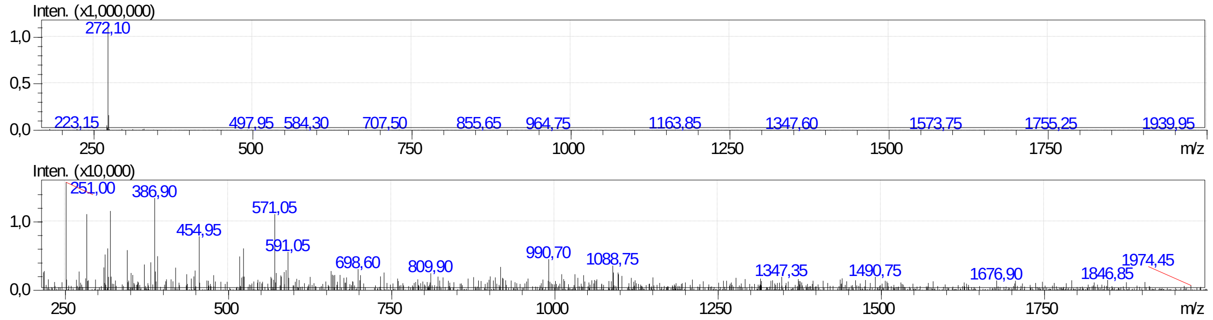

Peak# 4

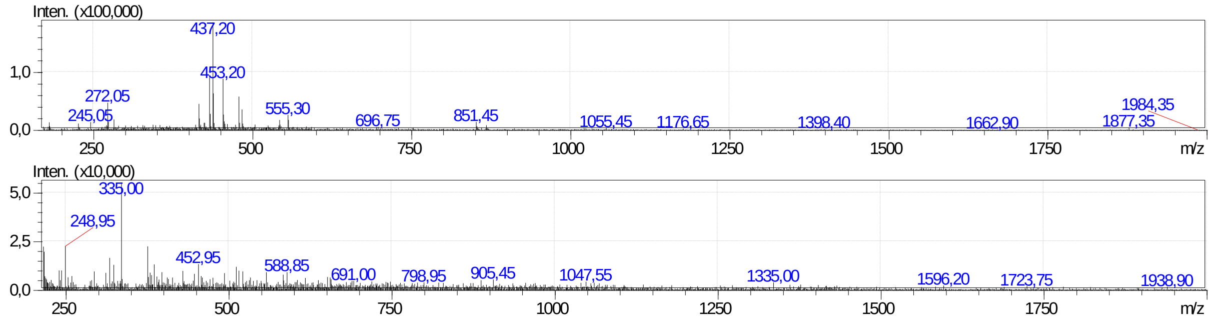

Polarity Negative ion

Peak# 1

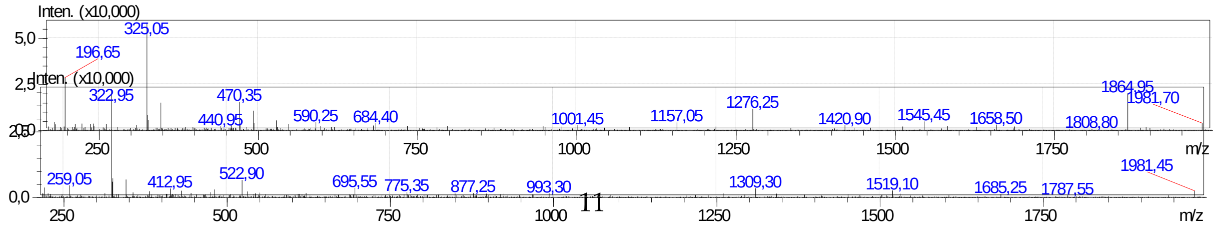

Peak# 2

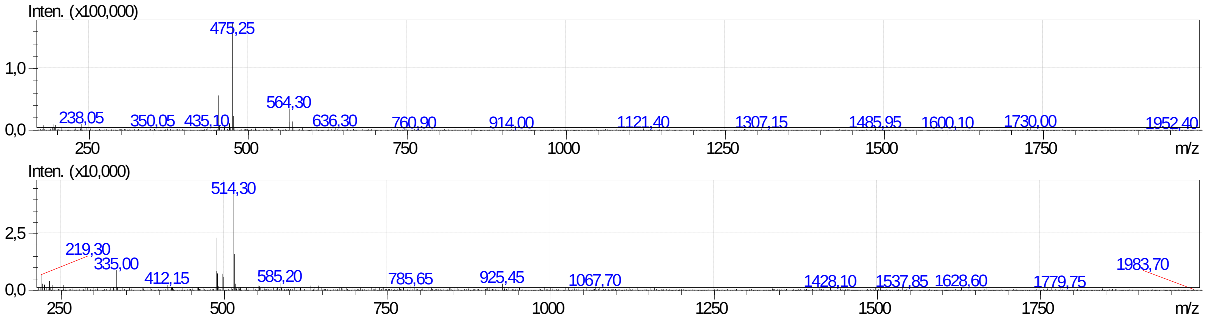

Peak# 3

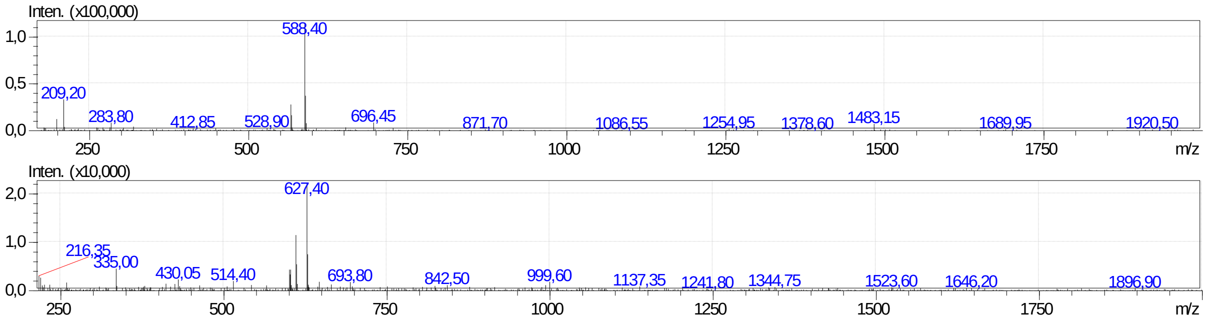

Figure S8. LC-MS mass spectra of the supernatant obtained in the reaction of Cu-NP with NAC (1:1 molar ratio).

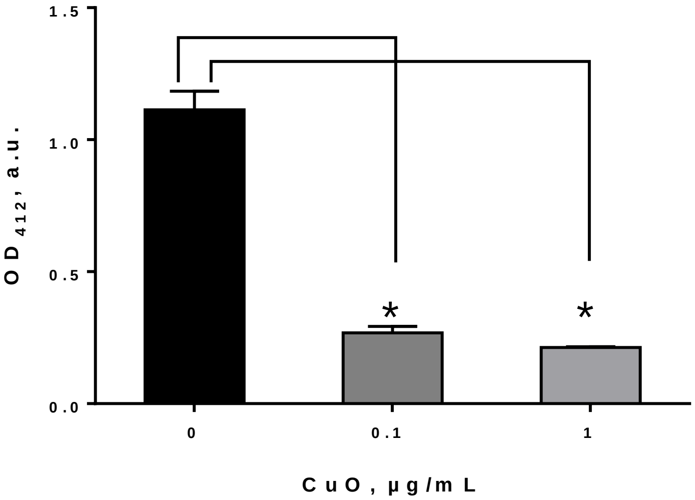

Figure S9. CuO abrogates the detection of SH groups with DNTB. Each sample contained 1 mM NAC. SH groups were detected in a colorimetric reaction (see *Experimental* for details). Note that the addition of CuO NPs immediately dropped OD values indicating a dramatic decrease of SH content. Shown are mean + SD of three independent measurements. *p<0.01.

Chromatogram

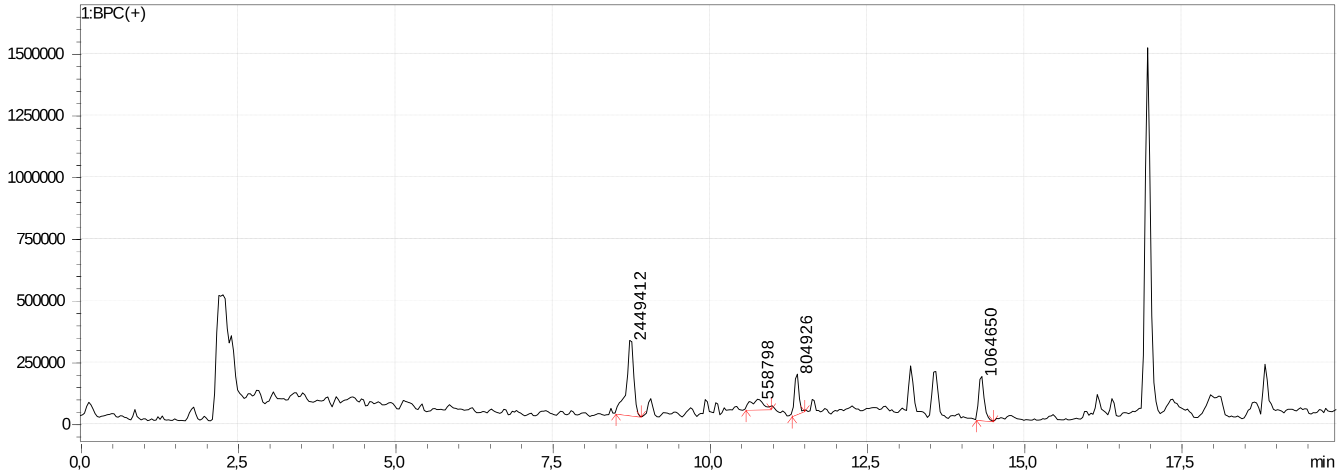

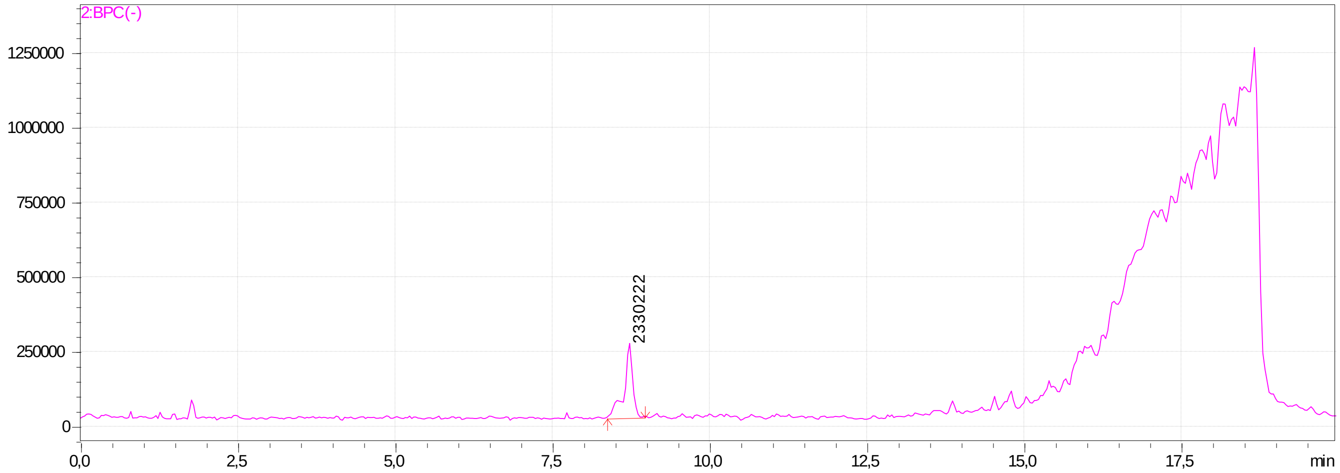

Polarity	Positive ion			Polarity	Negative ion		
Peak#	Ret. Time	Base Peak m/z	Area%	Peak#	Ret. Time	Base Peak m/z	Area%
1	8,749	325,05	50,216	1	8,727	323	100
2	10,775	178,1	11,456				
3	11,389	274,3	16,502				
4	14,32	437,15	21,826				

Spectra

Polarity Positive ion

Peak# 1

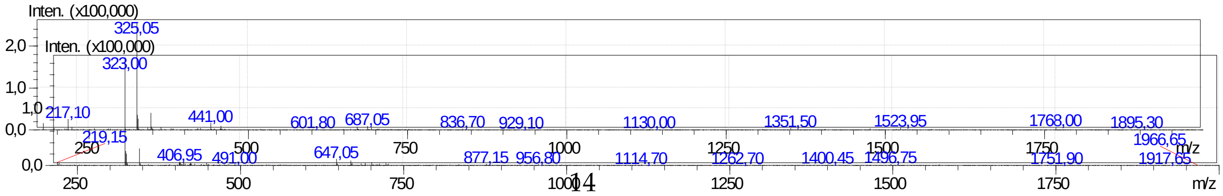

Peak# 2

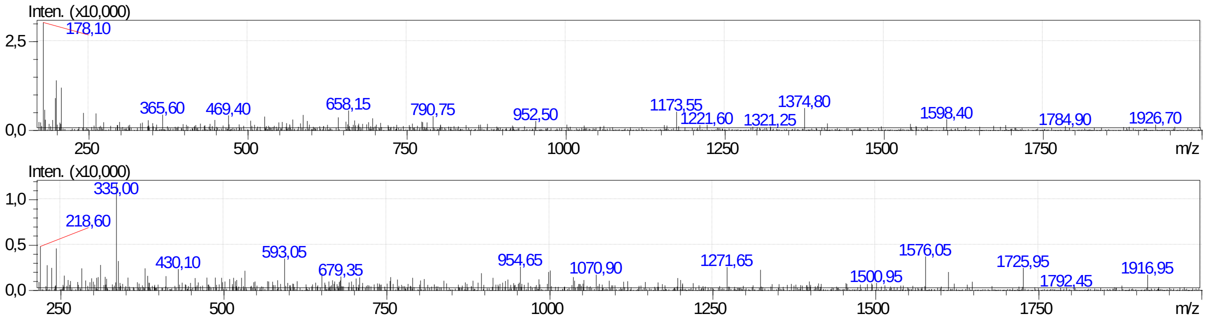

Peak# 3

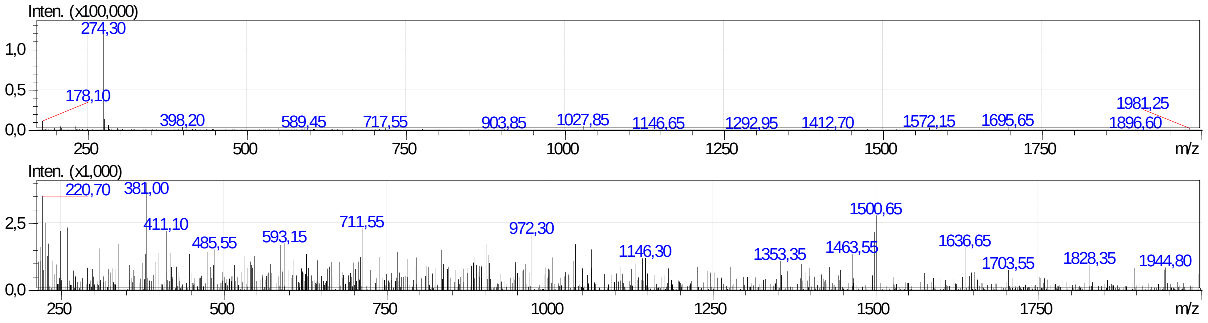

Peak# 4

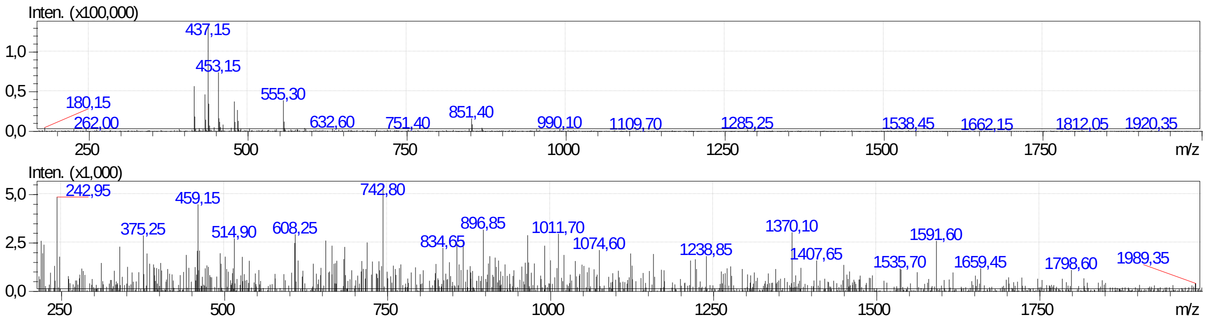

Polarity Negative ion

Peak# 1

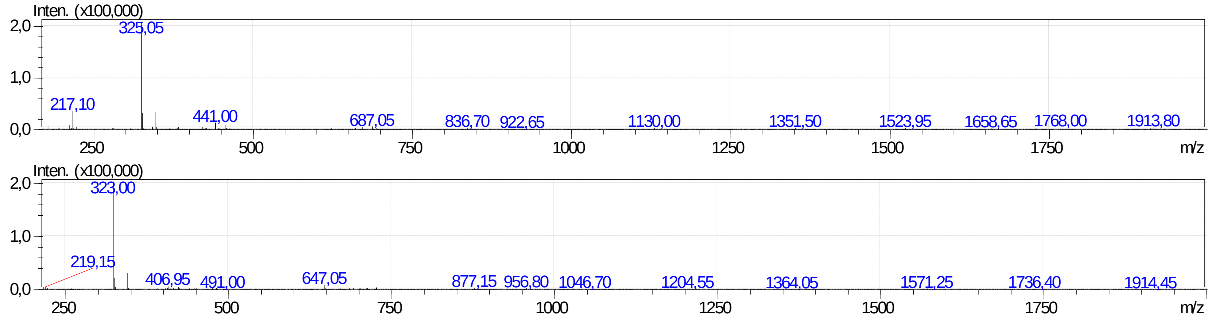

Figure S10. LC-MS mass spectra of the supernatant obtained in the reaction of Cu-NP with NAC (1:3 molar ratio).

Chromatogram

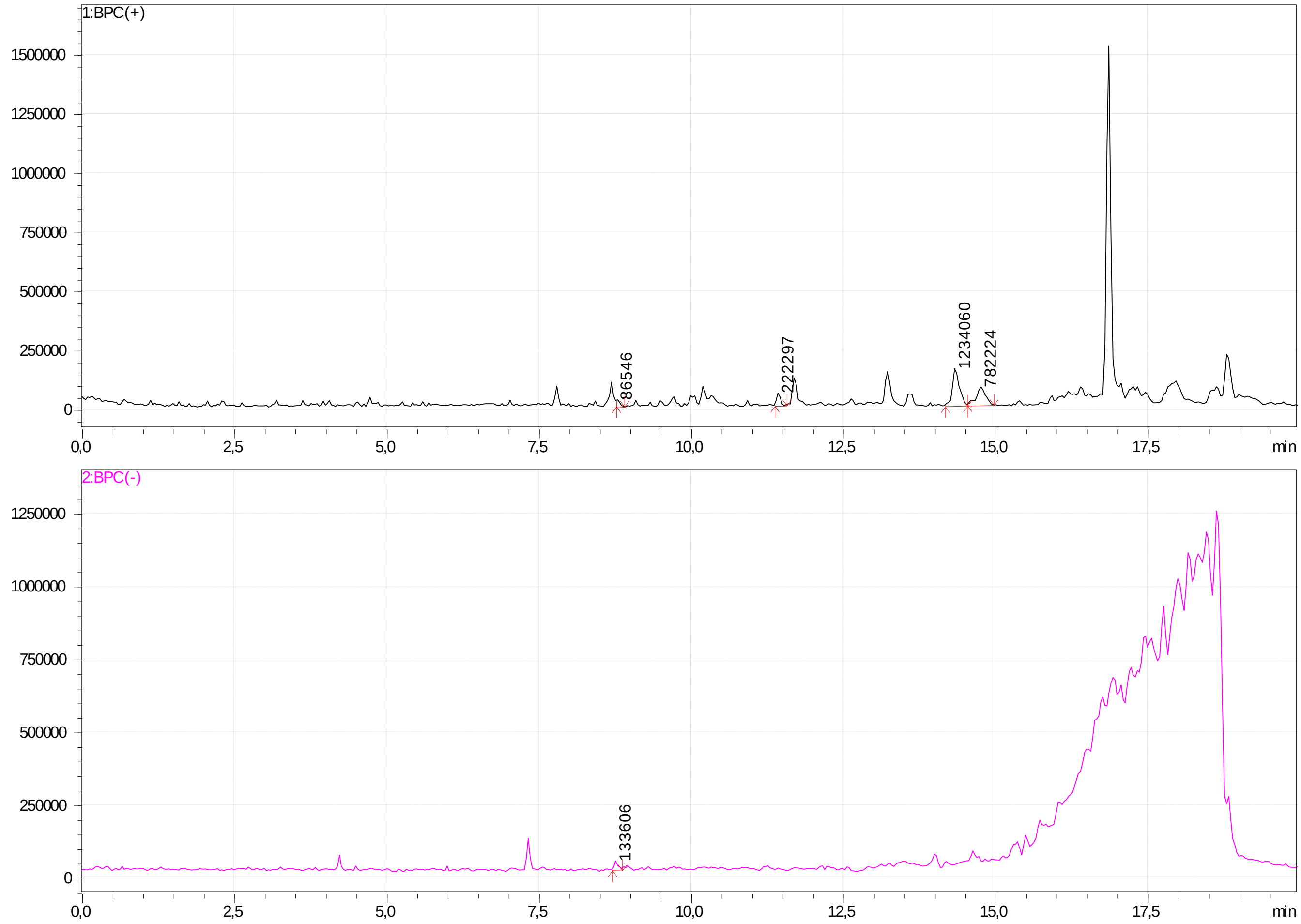

Polarity	Positive ion			Polarity	Negative ion		
Peak#	Ret. Time	Base Peak m/z	Area%	Peak#	Ret. Time	Base Peak m/z	Area%
1	8,79	325,05	3,722	1	8,77	323,05	100
2	11,439	274,25	9,561				
3	14,343	437,2	53,075				
4	14,766	437,2	33,642				

Spectra

Polarity Positive ion

Peak# 1

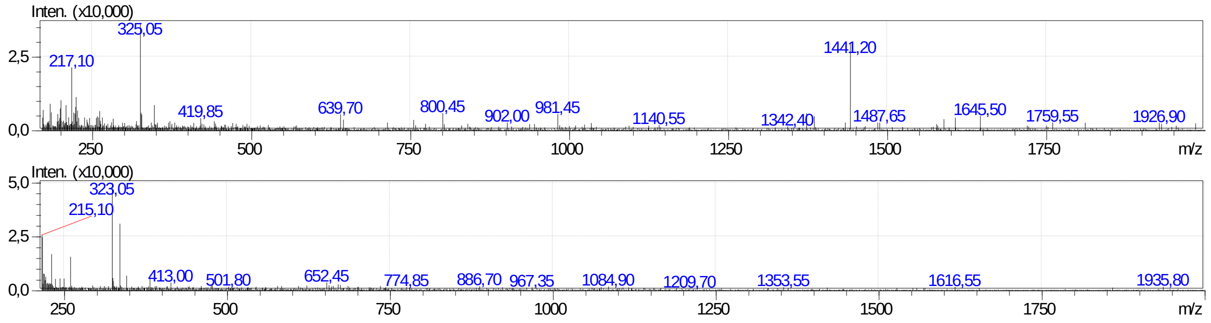

Peak# 2

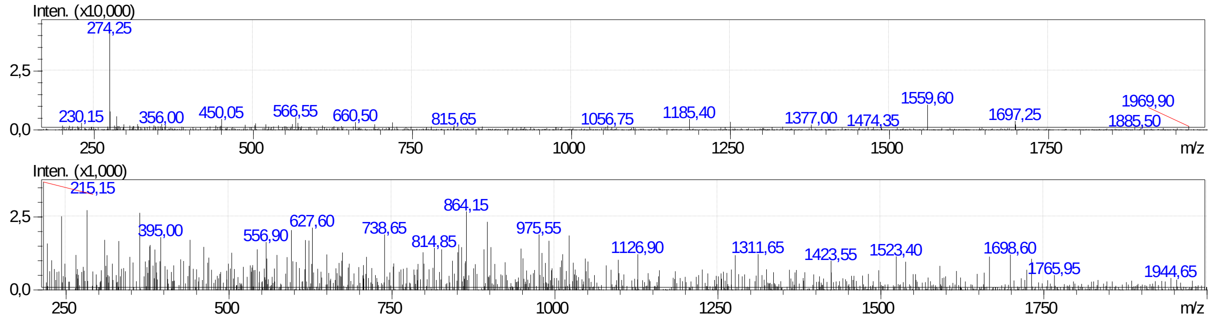

Peak# 3

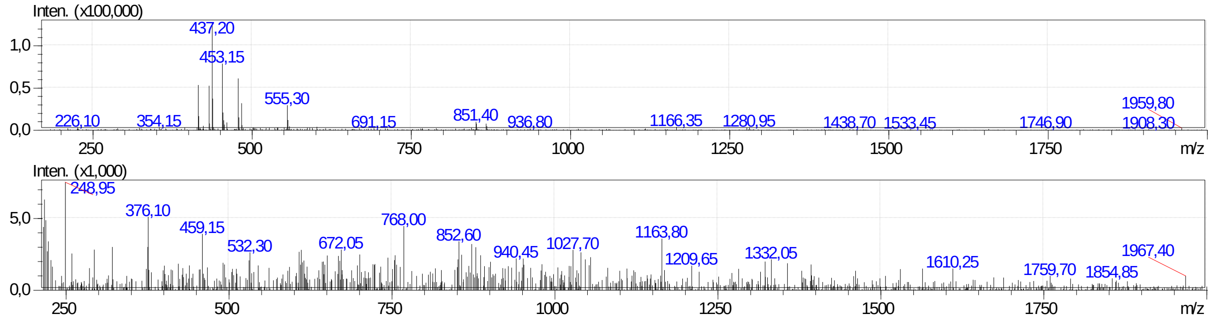

Peak# 4

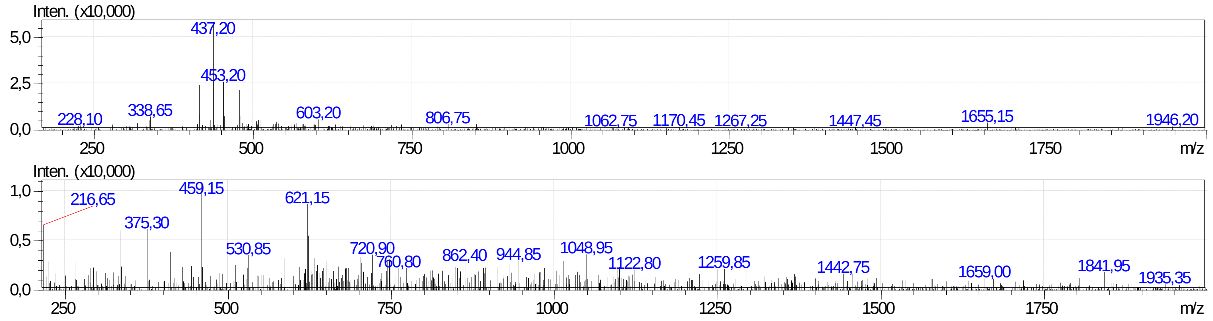

Polarity Negative ion

Peak# 1

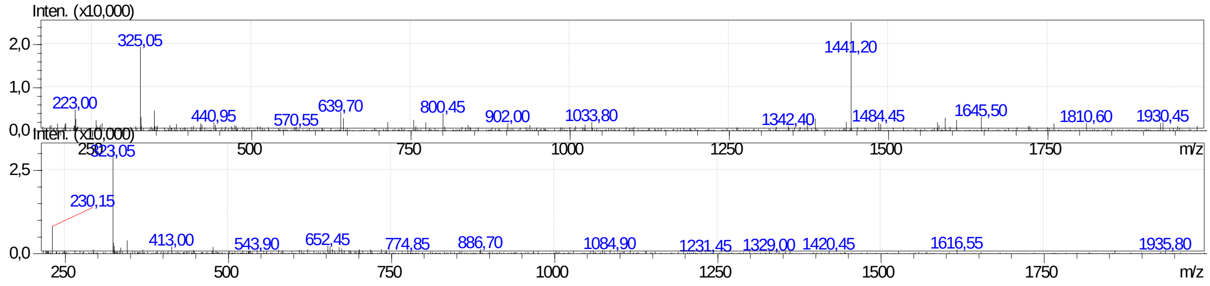

Figure S11. LC-MS mass spectra of the supernatant mixture obtained by the reaction of Cu-NP with NAC (1:10 molar ratio).

Table S1. Thermodynamic characteristics of DPPC and DPPG melting in the presence of NAC, CuO NPs and their combinations.

Treatment	Concentration	DPPC		DPPG	
		T_m, °C	$T_{1/2}$, °C	T_m, °C	$T_{1/2}$, °C
No	–	41.2	0.6	41.1	0.7
NAC	1 mM	41.2	0.6	41.7*	0.9*
CuO	1 μg/ml	41.2	0.6	41.1	0.7
	2 μg/ml	41.2	0.6	41.1	0.7
	4 μg/ml	41.2	0.6	41.1	0.7
NAC + CuO	1 mM + 1 μg/ml	41.2	0.6	42.5*	3.6*
	1 mM + 2 μg/ml	41.3	0.6	42.6*	3.0*
	1 mM + 4 μg/ml	41.3	0.6	42.6*	2.8*

Tm is maximum temperature of main lipid phase transition; *T1/2* is the half-width of the main peak. *deconvolution of the main peak is observed.
